# Fuzzy Tandem Repeats Containing p53 Response Elements May Define Species-Specific p53 Target Genes

**DOI:** 10.1371/journal.pgen.1002731

**Published:** 2012-06-28

**Authors:** Iva Simeonova, Vincent Lejour, Boris Bardot, Rachida Bouarich-Bourimi, Aurélie Morin, Ming Fang, Laure Charbonnier, Franck Toledo

**Affiliations:** 1 Institut Curie, Centre de Recherche, Paris, France; 2 UPMC Univ Paris 06, Paris, France; 3 CNRS UMR 3244, Paris, France; Stanford University School of Medicine, United States of America

## Abstract

Evolutionary forces that shape regulatory networks remain poorly understood. In mammals, the Rb pathway is a classic example of species-specific gene regulation, as a germline mutation in one *Rb* allele promotes retinoblastoma in humans, but not in mice. Here we show that p53 transactivates the *Retinoblastoma-like 2* (*Rbl2*) gene to produce p130 in murine, but not human, cells. We found intronic fuzzy tandem repeats containing perfect p53 response elements to be important for this regulation. We next identified two other murine genes regulated by p53 *via* fuzzy tandem repeats: *Ncoa1* and *Klhl26*. The repeats are poorly conserved in evolution, and the p53-dependent regulation of the murine genes is lost in humans. Our results indicate a role for the rapid evolution of tandem repeats in shaping differences in p53 regulatory networks between mammalian species.

## Author Summary

*TP53*, the gene encoding p53, is mutated in more than half of human cancers. Consequently, p53 is one of the most studied transcription factors, shown to directly regulate more than 150 genes. The mouse is a model of choice to study p53 mutants and cancer. However, differences were found between tumorigenesis in mice and humans, and these should be investigated to improve the relevance of mouse models. The distinct mutational events required to initiate retinoblastomas in these species constitute a classic example of such differences. Here we show that p53 regulates the *Retinoblastoma-like 2* (*Rbl2*) gene, encoding tumor suppressor p130, in murine but not human cells. The p53-dependent regulation of murine *Rbl2*/p130 relies on clustered p53 response elements, located within tandem repeats poorly conserved in evolution. A similar situation was found for two other genes, also p53 targets in mice but not in humans. Thus, tandem repeats may shape differences in mammalian p53 regulatory networks. By uncovering differences in p53 target gene repertoires between mice and humans, our findings may help to improve mice as models of human disease. In addition, the role of tandem repeats in shaping the target gene repertoires of other mammalian transcription factors should be considered.

## Introduction

Retinoblastoma is the most common pediatric intraocular tumor, that may arise in an unilateral or bilateral form. In human bilateral retinoblastoma, a germline mutation in one *RB1* allele is typically observed, and the second allele undergoes somatic mutation, in agreement with Knudson's «two-hit hypothesis» for inherited cancer syndromes [1]. This defined *RB* as the prototypical tumor suppressor gene, and prompted several groups to develop *Rb* mutant mice. However, *Rb* ^+/−^ mice were not found to develop retinoblastomas, but rather pituitary and thyroid tumors [2]–[4]. Retinoblastomas were observed in mutant mice lacking both Rb and the Rb-like protein p107, or both Rb and the Rb-like protein p130 [5]–[8]. Additionnal studies suggested that Rb loss in the mouse retina does not lead to retinoblastoma due to a compensatory upregulation of p107 and a partially redundant expression of Rb and p130 [9]–[12].

Inactivation of the p53 pathway plays an important role in the development of murine and human retinoblastomas [13], [14]. We were intrigued by reports showing that p107-deficient mice with Rb deletion in the developing retina (*Chx10Cre*; *Rb* ^lox/−^; *p107* ^−/−^) develop non invasive retinoblastomas with low penetrance, whereas similar mice with an additional retina-specific loss of p53 (*Chx10Cre*; *Rb* ^lox/−^; *p107* ^−/−^; p53^Lox/−^), or with decreased p107/p130 levels (*Chx10Cre*; *Rb* ^lox/lox^; *p107* ^+/−^; p130^−/−^), develop aggressive and invasive bilateral retinoblastomas [12], [13]. This led us to investigate whether p130 levels might be regulated by p53 in mice. Surprisingly, the results we obtained revealed common features for a subset of p53 target genes that are differentially regulated between mammalian species. We identified three genes (*Rbl2*/p130, *Ncoa1* and *Klhl26*) that are regulated in murine cells *via* clustered p53 response elements located within imperfect tandem repeats (often called Fuzzy Tandem Repeats or FTRs). Because the DNA sequence of FTRs is poorly conserved in evolution, the p53-dependent regulation of these genes is not observed in human cells, and only partially conserved in rat cells. These results provide insights into the evolution of p53 regulatory networks, which may help to understand species-specific differences in tumorigenesis.

## Results

### Murine *Rbl2*/p130 is a p53 target gene

**Figure 1:**
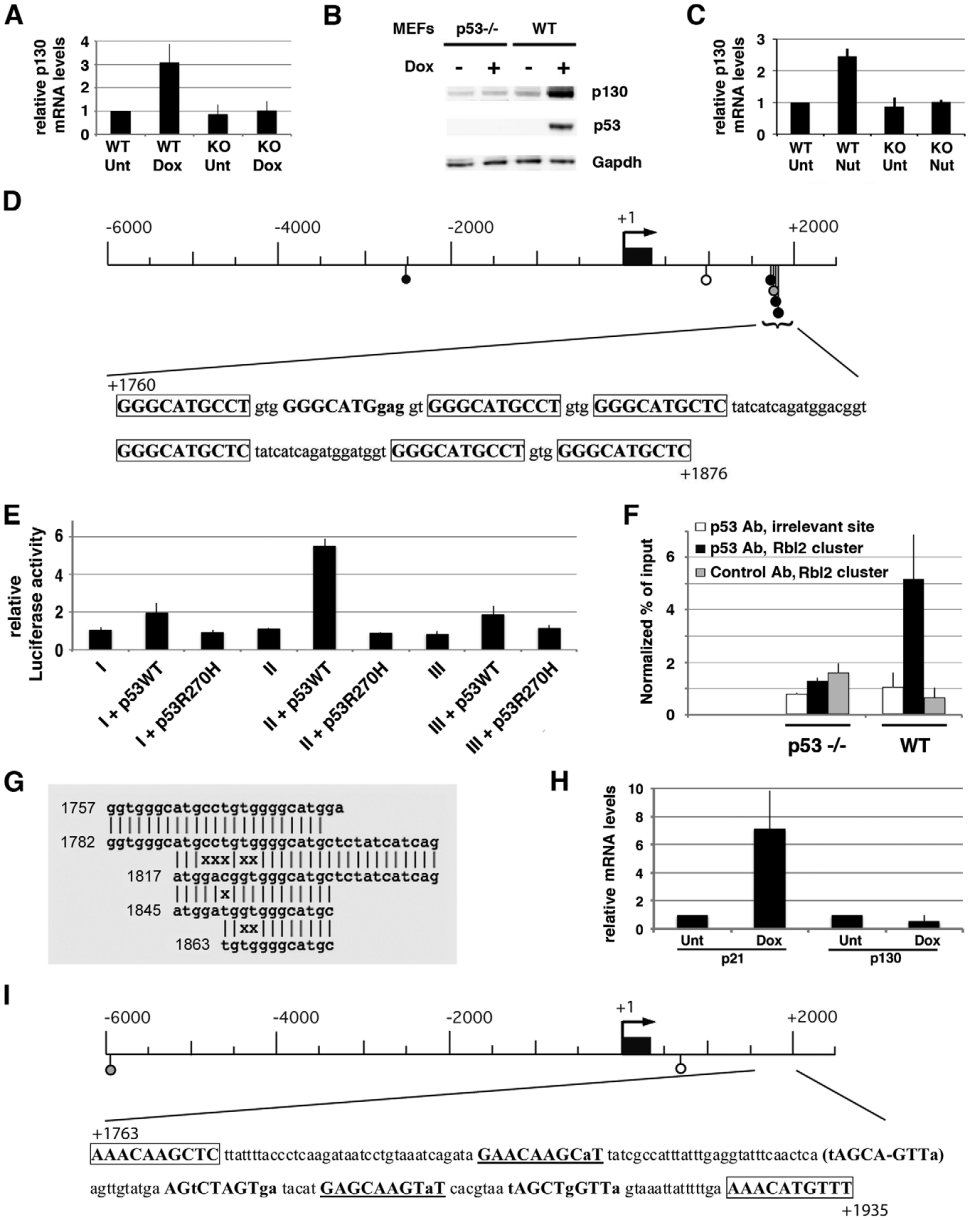
Murine *Rbl2/*p130 is a p53 target gene. (A) WT and p53^−/−^ MEFs were left untreated (Unt) or treated with doxorubicin (Dox) before RNA extraction and real-time PCR quantification, in 8 independent experiments. Data were normalized to control mRNA levels, then a value of 1 was assigned to mRNA amounts in unstressed WT cells. (B) MEFs were treated as in (A), then protein extracts were immunoblotted with antibodies to p130, p53 and Gapdh. (C) MEFs were left untreated (Unt) or treated with Nutlin (Nut) before RNA quantification, in 4 independent experiments. (D) Putative p53 REs, identified using Consite and a positional frequency matrix (Methods), were plotted along the *Rbl2*/p130 locus as lollipops, with greytones according to scores (Table S1). Numbers are relative to the transcription start site (TSS). Black box: exon 1. Below, the cluster sequence is shown, with p53 putative binding half-sites in bold (putative binding sites have 0–3 mismatches with the consensus; matches in capital letters and mismatches in lowercase; perfect half-sites are boxed). (E) The 6 kb upstream the TSS were cloned before a luciferase reporter gene, and the plasmid was transfected in p53^−/−^ MEFs alone (*I*), or with an expression vector for WT p53 (*I*+p53WT) or mutant p53 (*I*+p53R270H) to measure luciferase. Likewise, luciferase was measured with plasmids containing 2.5 kb of sequences downstream the TSS with (*II*) or without (*III*) the clustered p53 REs. Results, from 3 independent experiments, were normalized to control renilla luciferase, then a value of 1 was assigned to luciferase in cells transfected with reporter plasmids alone. (F) ChIP assay was performed in doxorubicin-treated MEFs, with an antibody against p53, or rabbit IgG as a control. Immunoprecipitates were quantified using real-time PCR and normalized to input DNA on an irrelevant region, in 3 independent experiments. (G) Integrated results of the cluster sequence analysis with *mreps*. Numbers are relative to the TSS. (H) Human lung fibroblasts were treated and mRNAs were quantified as in (A), in 3 independent experiments. Similar results were obtained with foreskin fibroblasts. (I) Putative p53 response elements were searched for and plotted along the human *Rbl2*/p130 locus as in (D). The region homologous to the murine clustered p53 REs is below, with putative half-sites as before (those with a single mismatch are underlined). The parentheses indicate a nonamer that might be a putative half-site with a deletion within the core CWWG motif (the minus sign indicates the deletion), a situation observed in about 5% of p53 binding half-sites [41].

Consistent with the hypothesis that murine p130 could be regulated by p53, we observed an increase in p130 mRNA levels in response to doxorubicin in wild-type, but not p53^−/−^ mouse embryonic fibroblasts (MEFs) (Figure 1A). A similar observation was obtained *in vivo*, in tissues from irradiated mice (Figure S1). Importantly, the stress-dependent increase in p130 mRNA levels led to an increase in p130 protein levels in WT MEFs (Figure 1B). We also found an increase in p130 mRNA levels in WT cells treated with Nutlin, the specific inhibitor of MDM2-p53 interaction [15], further suggesting that the stress-dependent induction of p130 results from an increased p53 activity (Figure 1C).

We next used an *in silico* approach to search for potential p53 binding sites in the murine *Rbl2* gene. The consensus sequence for a p53 response element (p53 RE) was first defined as 2 copies of the 10-base pair motif RRRCWWGYYY separated by 0–13 base pairs (where R = G/A, W = A/T and Y = C/T) [16]. More recently, genome-wide ChIP-on-chip experiments allowed to further refine the consensus sequence (reviewed in [17], [18]). To perfom an unbiased search for p53 putative binding sites, we used a positional frequency matrix (PFM) recently generated by a genome-wide ChIP-on-chip experiment in human cells [19], modified to take into account varying spacer lengths (Figure S2 and Methods). An analysis of sequences from 6 kb upstream to 2.5 kb downstream of the p130 mRNA transcription start site (TSS) suggested one putative p53 RE 2.5 kb upstream of the TSS but more strikingly, a distinctive 117 bp-long cluster of putative p53 REs containing 7 p53 binding half-sites, 6 of which perfectly match the canonical RRRCWWGYYY motif (Figure 1D and Table S1). When the sequences upstream of the TSS were cloned before a luciferase reporter gene, little p53-dependent induction could be measured, whereas a strong p53-dependent induction was found for sequences downstream of the TSS, and the deletion of clustered p53 REs strongly reduced this induction (Figure 1E). In addition, the cluster of p53 REs alone, when cloned before a luciferase reporter gene, ensured a strong p53-dependent induction (Figure S3). Chromatin immunoprecipitation experiments in stressed WT and p53^−/−^ cells then indicated significant binding of p53 to this cluster *in vivo* (Figure 1F). Altogether, the results show that p53 transactivates murine *Rbl2*/p130, and that a cluster of p53 REs in intron 1 is important for this regulation.

### The p53 REs in murine *Rbl2* are within fuzzy tandem repeats

The cluster of p53 REs in the murine *Rbl2* gene has several unusual features: 1) a clustering of p53 binding half-sites has already been described for a few p53 target genes, but the proposed rule for such clustering is that two p53 binding half-sites match the consensus RRRCWWGYYY sequence almost perfectly, and additionnal half-sites have more degenerated sequences [20]. In the murine *Rbl2* gene, 6 out of 7 p53 half-sites are perfect matches to the consensus, thus exceeding by far the proposed criteria for clustered p53 binding half-sites; 2) transactivation was shown to occur through p53 binding to repeated sequences such as retroviral elements for a fraction of p53 target genes [21], [22], or to an unusual microsatellite repeat (TGYCC)_n_ for the target gene *PIG3* [23]. A duplication most likely participated in the creation of the cluster in murine *Rbl2*, as evidenced by the sequence similarities between its two larger spacers (Figure 1D). However the cluster, as a whole, does not correspond to any previously described repeated sequence. To better understand the structure of the cluster, we used *mreps*, a software designed to detect repeats, even if they are «fuzzy», i.e. if they contain mismatches [24]. Strikingly, *mreps* detected fuzzy tandem repeats encompassing the entire cluster of p53 REs in murine *Rbl2* (Figure 1G and Figure S4).

### The p53 putative binding sites are mutated in human *Rbl2*

In human cells, several reports indicated that p130 is involved in the p53–p21 damage response leading to cellular senescence, but that p53 does not transactivate *Rbl2*/p130 [25], [26]. However, the classical assumption that important regulatory networks are evolutionnary conserved is now being challenged, particularly for trancription factors Oct4 and Nanog [27], but also for p53 [21], [22], [28]. Consistent with previous reports, we observed that, unlike the well-known p53 target *Cdkn1a*/p21, *Rbl2*/p130 is not induced in normal human fibroblasts after treatment with doxorubicin (Figure 1H) or Nutlin (not shown). The apparent difference in *Rbl2* regulation between murine and human cells led us to analyze the nucleotide sequence of the human *Rbl2* gene. No cluster of putative p53 RE was found in the intron 1 of human *Rbl2* when using the same PFM-based criteria as for the murine locus, and most p53 putative binding half-sites were degenerated in the region homologous to the clustered p53 REs in the murine gene (Figure 1I and Table S1). Accordingly, *mreps* did not detect fuzzy tandem repeats in the intron 1 of human *Rbl2*. Thus, this intronic *Rbl2* region is poorly conserved between the murine and human genomes.

### BLAST with the *Rbl2* p53 RE cluster reveals another murine-specific p53 target

**Figure 2:**
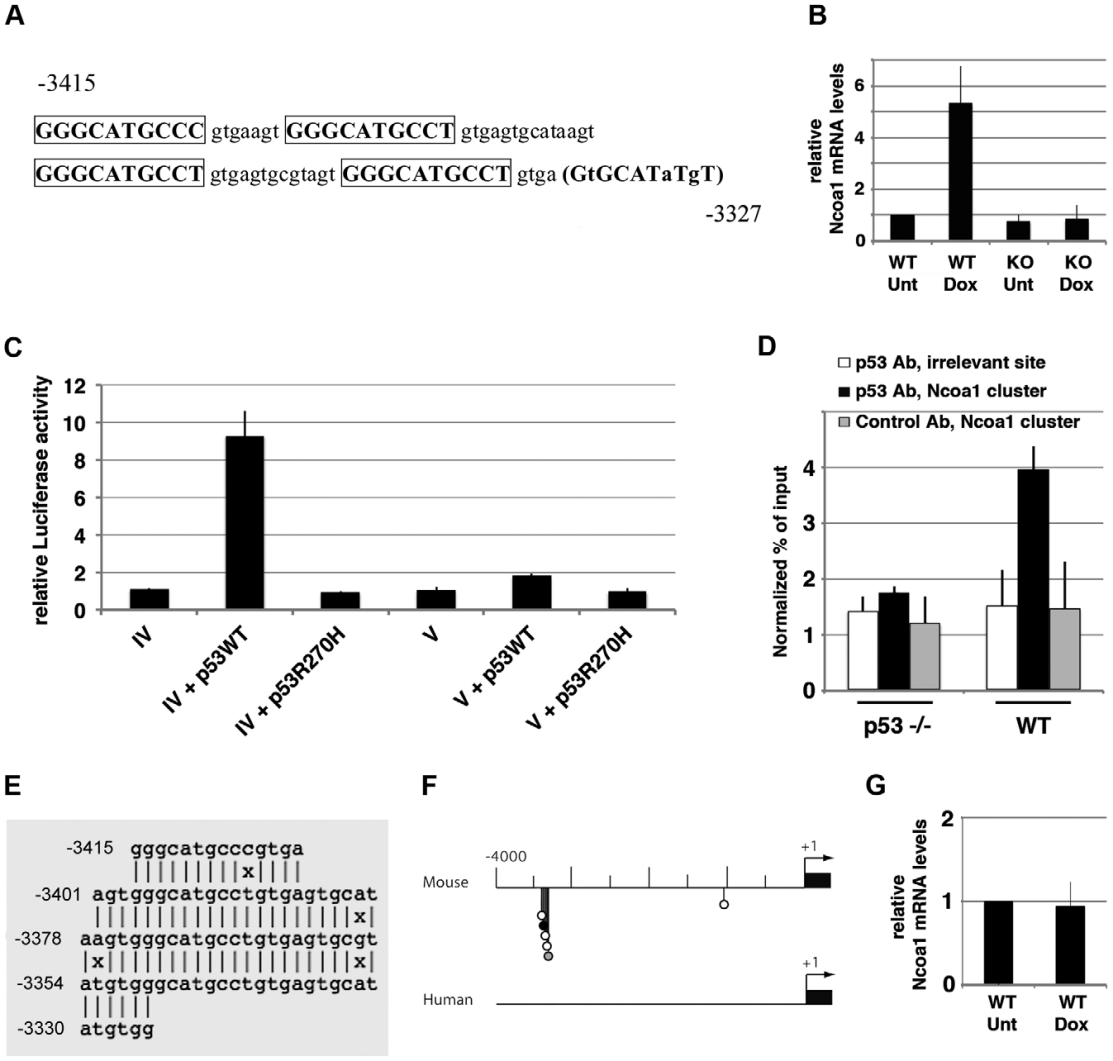
Transactivation of murine *Ncoa1* by p53 relies on a cluster of p53 half-sites. (A) Sequence of the clustered p53 REs upstream of the *Ncoa1* gene, represented as in Figure 1D. Numbers are relative to the TSS. The parentheses indicate a putative half-site with a rare variant of the core CWWG motif: evidence that p53 may bind a half-site with a CWWA core was reported [42], but only 2% of the sequences bound by p53 contain a p53RE with a CWWA core in its second half-site [19]. (B) MEFs were left untreated (Unt) or treated with doxorubicin (Dox) for 24 h, before RNA extraction and real-time PCR quantification. Results, from 7 experiments, were plotted as in Figure 1A. (C) A 2.5 kb-long fragment containing sequences upstream of the Ncoa1 TSS (from −3.5 kb to −1 kb) was cloned before a luciferase reporter gene, and transfected in p53^−/−^ MEFs alone (*IV*), or with an expression vector for WT p53 (*IV*+p53WT), or mutant p53 (*IV*+p53R270H), then luciferase activities were measured. Luciferase activities were determined with the same sequences after deletion of the clustered putative p53 REs (sequences from −3.1 kb to −1 kb; plasmid *V*). Results, from 3 independent experiments, were normalized to control renilla luciferase, then plotted as in Figure 1E. (D) ChIP assay was performed in doxorubicin-treated p53^−/−^ and wild-type MEFs, with an antibody against p53 or rabbit IgG as a control. Immunoprecipitates, from 3 independent experiments, were quantified and plotted as above. (E) Fuzzy tandem repeats within the *Ncoa1* cluster. Integrated *mreps* results are represented as in Figure 1G. (F) The cluster of p53 REs at the *Ncoa1* locus is poorly conserved in evolution. Candidate p53 REs were searched for at the murine and human *Ncoa1*loci and plotted as before. (G) Human fibroblasts were left untreated (Unt) or treated with doxorubicin (Dox) for 24 h, before RNA extraction and real-time PCR quantification. After stress, p21 mRNAs levels increased (Figure 1H), but not Ncoa1 mRNA levels. Results, from 3 independent experiments, were normalized and plotted as before.

We performed a BLAST (basic local alignment search tool) over the mouse genome with the murine *Rbl2* p53 RE cluster to determine if similar cluster(s) could be involved in the regulation of other p53 target gene(s). A gene was considered a candidate p53 target if stress-induced in WT but not p53^−/−^ MEFs (with an induction at least 2-fold in WT cells), and if containing a cluster of p53 binding half-sites less than 10 kb upstream, or less than 5 kb downstream, of the TSS. Only one candidate gene was found to fulfill these criteria: *Ncoa1* (also known as *SRC1*), with a cluster of p53 putative half-sites 3.4 kb upstream of the TSS (Figure 2A, 2B and Figure S5). Further sequence analysis of this cluster with the p53 PFM and Consite suggested 5 candidate p53 REs (Table S2). Luciferase assays then demonstrated the importance of this cluster in the p53-dependent transactivation of *Ncoa1* (Figure 2C), and ChIP showed a modest, but significant p53 binding to this cluster *in vivo* (Figure 2D). Like for murine *Rbl2*, *mreps* identified fuzzy tandem repeats within the murine *Ncoa1* locus (Figure 2E, Figure S6), and the cluster was poorly conserved in evolution (Figure 2F). Accordingly, we did not observe a stress-dependent induction of *Ncoa1* in human fibroblasts (Figure 2G). A BLAST search using the cluster of p53 half-sites at the *Ncoa1* locus did not yield any additional candidate target gene.

### BLAST searches with synthetic clusters reveal a third gene regulated by p53 in murine but not human cells

**Figure 3:**
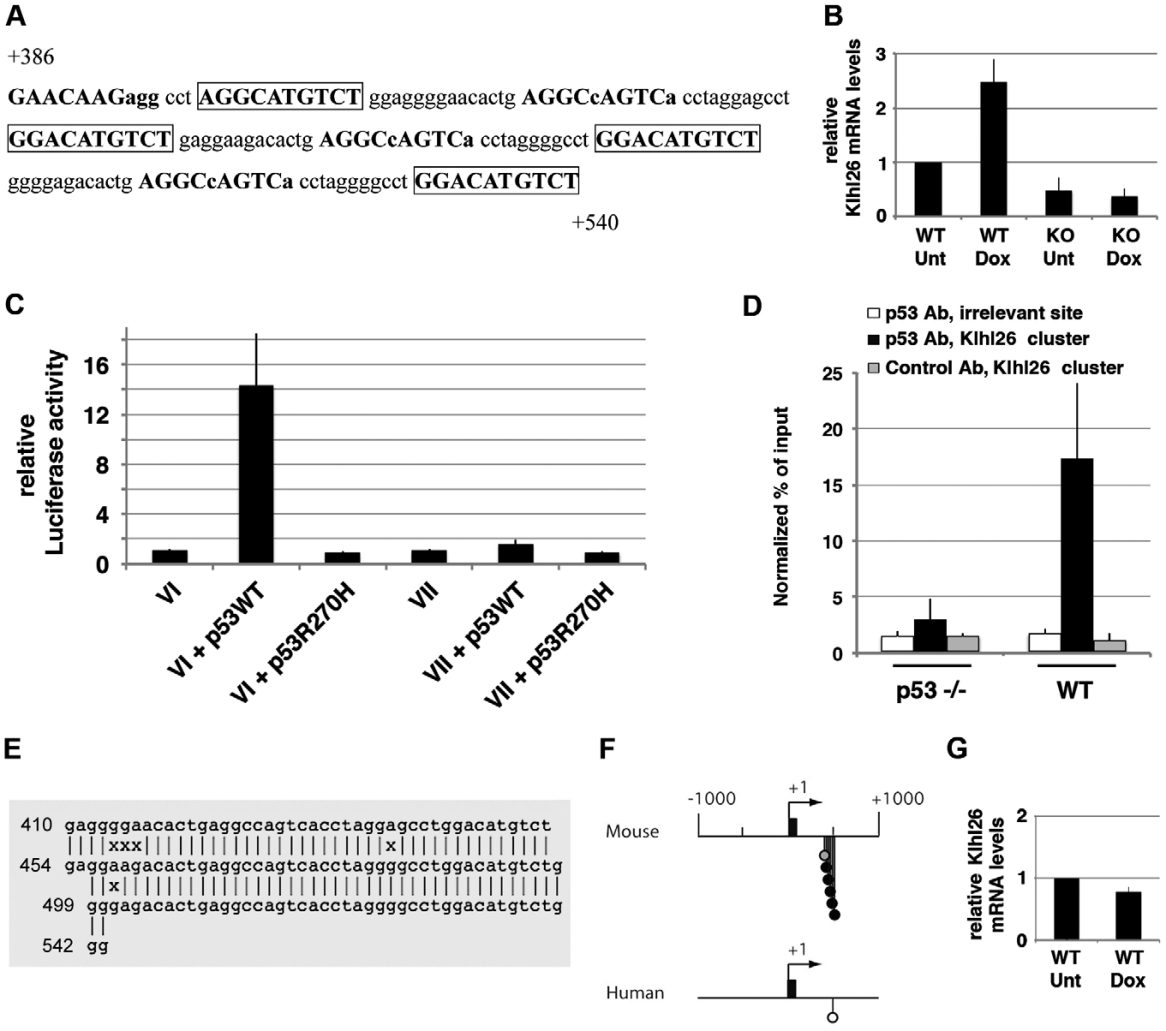
p53 transactivates murine *Klhl26* via a cluster of p53 half-sites. (A) Sequence of the cluster of p53 half-sites in *Klhl26* intron 1, represented as in Figure 1D. Numbers are relative to the TSS. (B) WT and p53^−/−^ MEFs were left untreated (Unt) or treated with doxorubicin (Dox) for 24 h, before RNA extraction and real-time PCR quantification. Results from 3 independent experiments. (C) A 1 kb-long fragment from *Klhl26* intron 1 (from +0.1 kb to +1.1 kb) was cloned before a luciferase reporter gene, to test for p53-dependent reporter activity. The reporter plasmid was transfected in p53^−/−^ MEFs alone (*VI*), together with an expression vector for p53 WT (*VI*+p53WT) or for mutant p53 (*VI*+p53R270H) and luciferase activities were measured. Similarly, luciferase activities were determined with the same sequences after deletion of clustered putative p53 half-sites (sequences from +0.39 to +0.64 kb were deleted; plasmid *VII*). Results, from 3 independent experiments, were normalized to control renilla luciferase, then plotted as in Figure 1E. (D) ChIP assay was performed in doxorubicin-treated p53^−/−^ and wild-type MEFs, with an antibody against p53 or rabbit IgG as a control. Immunoprecipitates, from 3 independent experiments, were quantified and plotted as before. (E) Integrated results of the *mreps* DNA sequence analysis, represented as in Figure 1G. (F) The cluster of p53 REs at the *Klhl26* locus is poorly conserved in evolution. Candidate p53 REs were searched for at the murine and human *Klhl26* loci and plotted as before. (G) *Klhl26* is not induced in response to stress in human fibroblasts. Human fibroblasts were left untreated (Unt) or treated with doxorubicin (Dox) for 24 h, before RNA extraction and quantification. After stress, p21 mRNAs levels increased (Figure 1H), but not Klhl26 mRNA levels. Results, from 4 independent experiments, were normalized and plotted as before.

We next decided to perform BLAST searches relying on the use of synthetic clusters of p53 REs. Each synthetic cluster was composed of 11 identical copies of a p53 RE, and each p53 RE had a sequence among the 128 most likely to be bound by p53, according to a genome-wide ChIP study [19] (Table S3). We performed 128 BLAST searches using synthetic clusters with the same criteria as before: a gene with a cluster 10 kb upstream or 5 kb downstream of its TSS, stress-induced at least 2-fold only in WT MEFs, was considered a candidate p53 transcriptional target. With this approach, an additional p53 target gene was identified: *Klhl26*. In a recent genome-wide ChIP-chip study, *Klhl26* was listed among 573 stress-induced genes bound by p53 in murine ES cells [29]. However the study focused on genes in the Wnt signalling pathway, so that neither luciferase assays, nor quantitative RT-PCR in WT and p53^−/−^ cells were performed to test if *Klhl26* and 500+ other candidates were indeed *bona fide* p53 transcriptional targets [29]. Here we show that *Klhl26* is a p53 target gene, and that the binding of p53 to clustered p53 REs is important for this regulation (Figure 3A–3D, Figure S5 and Table S4). Again, fuzzy tandem repeats are detected at the level of the cluster of p53 REs (Figure 3E, Figure S7), and the cluster is poorly conserved in evolution (Figure 3F–3G). BLAST searches with the cluster of p53 REs at the *Klhl26* locus did not suggest additional candidate target genes.

### Evidence for a partial divergence in the regulation of *Rbl2*, *Ncoa1*, and *Klhl26* among rodents

**Figure 4:**
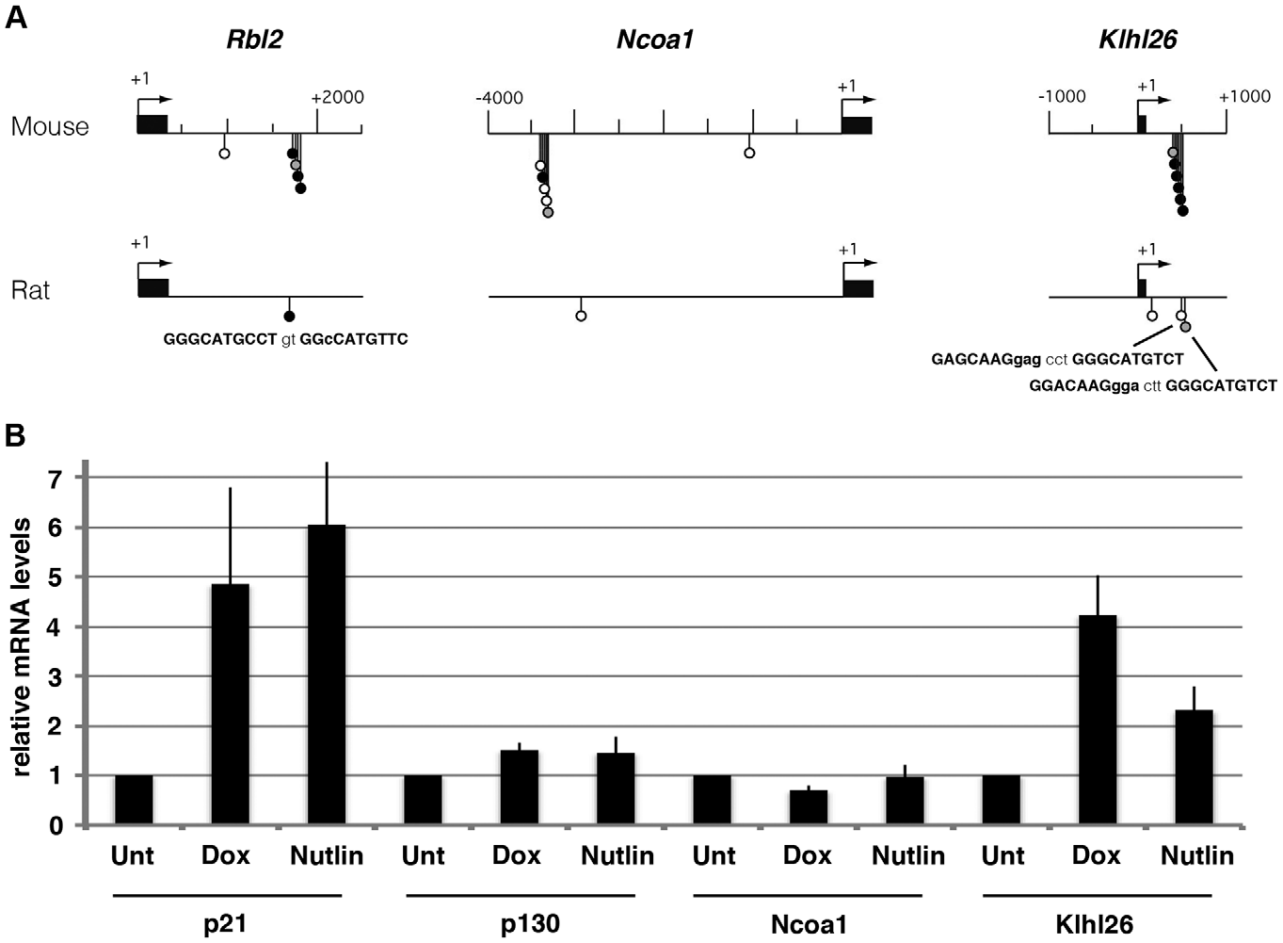
A partial divergence in the regulation of *Rbl2*/p130, *Ncoa1*, and *Klhl26* among rodents. (A) Homologous regions of the *Rbl2*, *Ncoa1* and *Klhl26* loci from mouse and rat were analyzed with Consite, and results were plotted along the map as before. At each rat locus, 0–2 putative p53 REs were found to map at the same position as a cluster of p53 REs at the homologous murine locus region. Their sequence is indicated below the map. (B) Primary REFs were left untreated (Unt) or treated for 24 h with doxorubicin (Dox) at 0.5 µg/ml, or Nutlin at 10 µM, before RNA extraction and real-time PCR quantification. Results were normalized to control mRNA levels, then the mean amount of mRNAs in unstressed cells was assigned a value of 1. Data are from 3 independent experiments.

We identified 3 genes that are p53 transcriptional targets in murine, but not human cells. To evaluate conservation among rodents, we analyzed the promoters of these 3 genes in the rat genome. A single putative p53 RE was found at the rat *Rbl2* locus at the same location as the cluster of p53 REs in the mouse gene, whereas 0 and 2 putative REs were respectively found at the rat *Ncoa1* and *Klhl26* loci, in the regions homologous to those containing clustered p53 REs in the murine genes (Figure 4A). These analyses indicated a significant divergence in the DNA sequence between the mouse and rat genomes at the 3 loci, with possible regulatory consequences. We next analyzed the mRNA levels for p21, p130, Ncoa1 and Klhl26 in wild-type primary rat embryonic fibroblasts (REFs), before or after treatment with doxorubicin or Nutlin. Both drugs led to strong increases in p21 and Klhl26 mRNAs, whereas p130 mRNA levels were barely increased, and Ncoa1 mRNAs were not increased at all (Figure 4B). From these results we conclude that the p53-dependent regulation of *Rbl2*, *Ncoa1* and *Klhl26* observed in murine cells is, at best, only partially conserved in rat cells.

### Sequences containing clustered p53 half-sites diverge rapidly

**Figure 5:**
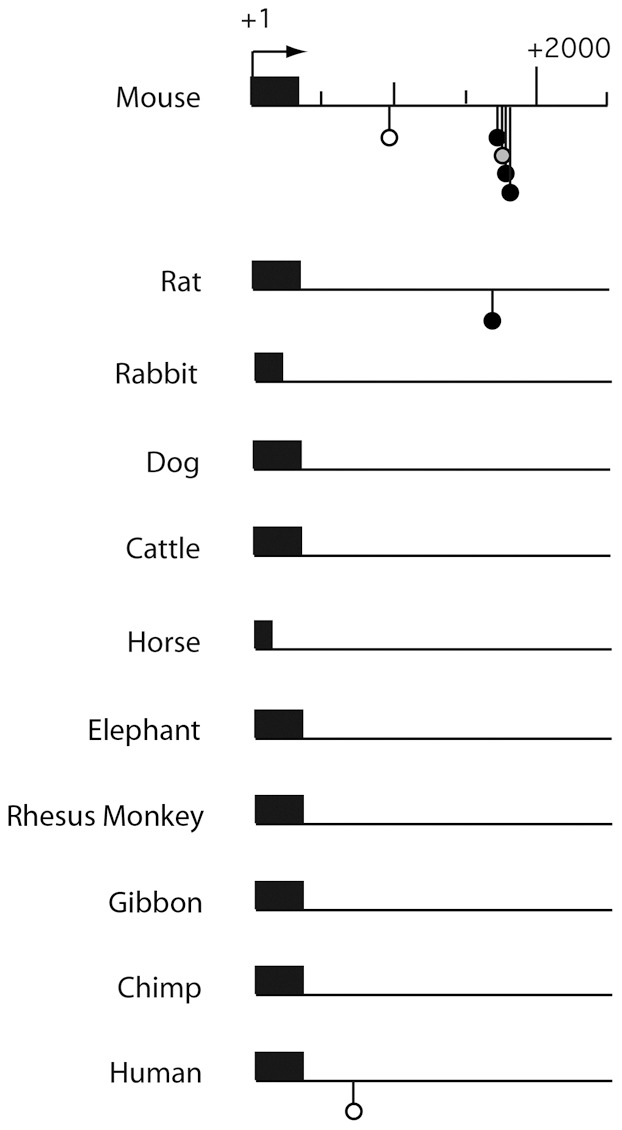
Comparative Consite analysis of *Rbl2* sequences from 11 mammalian species. The sequences for the first 2.5 kb downstream of the *Rbl2* transcription start site (mouse, rat, dog, cattle, rhesus monkey, gibbon, chimp, human) or translation start site (rabbit, horse, elephant) were analyzed using Consite as before. Results are represented as in Figure 1D.

**Figure 6:**
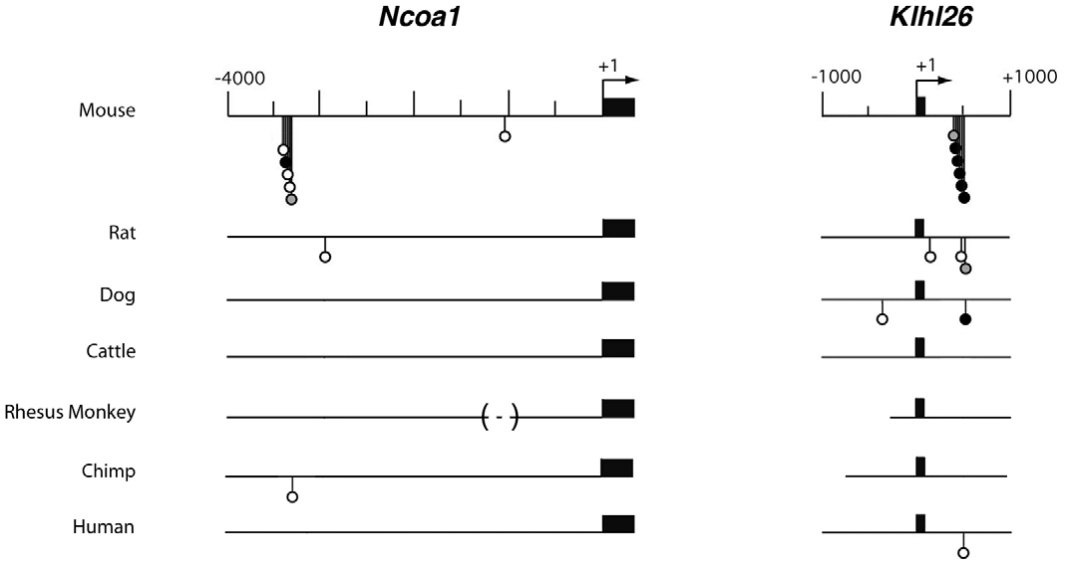
Comparative Consite analyses of *Ncoa1 and Klhl26* sequences from 7 mammalian species. The 4 kb of DNA sequences upstream of the *Ncoa1* transcription start site (left), or the 2 kb surrounding the *Klhl26* transcription start site (right), were analyzed in 7 mammalian species using Consite as before. Results are represented as in Figure 1D. Shorter lines, or dashed lines within brackets, indicate incomplete sequences.

We performed a Consite analysis of the first 2.5 kb of *Rbl2* genomic sequences in 8 additional mammalian species (rabbit, dog, cattle, horse, elephant, Rhesus monkey, gibbon and chimp): this provided further evidence that the clustered p53 REs in murine *Rbl2* are poorly conserved in evolution (Figure 5). Consistent with this, when we searched for sequences containing clustered p53 half-sites in these 8 species in addition to mouse, rat and human, we identified a partial conservation between rodent species, or among primates, but sequences were more divergent when distant species were compared (Figure S8). Likewise, the Consite analysis of genomic sequences at the *Ncoa1* and *Klhl26* loci in 7 mammalian species indicated a divergence in regulation among mammals (Figure 6), further supported by a search for sequences containing clustered putative p53 half-sites. At the *Klhl26* locus, a partial conservation was found among rodents or among primates, with significant divergence between more distant species (Figure S9). At the *Ncoa1* locus, we could not identify significant conservation even between closely related species (not shown). These results indicate that tandem repeats containing p53 response elements evolve rapidly, which may account for differences between mammalian p53 transcriptional repertoires.

## Discussion

Our analysis of species-specific gene regulation started from the observation that unlike humans, *Rb* ^+/−^ mice do not develop retinoblastomas [2]–[4]. Instead, retinoblastomas develop in mice with a concomitant loss of Rb and the Rb-like protein p107, or a concomitant loss of Rb and the Rb-like protein p130 [5]–[8]. Here we identified *Rbl2*/p130 as a p53 target gene in mouse, but not human cells (Figure 1). Thus, like *Rbl1*/p107 [9]–[12], *Rbl2*/p130 is differentially regulated in mice and humans, which may account for the different mutational events required to initiate retinoblastoma in these species. Indeed, our findings may explain why aggressive bilateral retinoblastomas develop after a retina-specific deletion of Rb in mice with decreased p107/p130 levels, or a retina-specific loss of both Rb and p53 in mice lacking p107, but not after a retina-specific loss of Rb in mice lacking only p107 [12], [13]. Also possibly consistent with our results, Rb loss is compensated by increased levels of both p107 and p130 in murine Ras-induced lung tumors, whereas in lung tumors initiated by a concomitant loss of Rb and p53, p107 levels are increased but p130 levels are decreased [30], [31]. Interestingly, murine lung tumors initiated by a loss of Rb and p53, and those initiated by the loss of Rb, p53 and p130, shared highly similar transcriptomes [31]. Together, these data suggest that the regulation of murine *Rbl2*/p130 by p53 may affect tumor initiation or progression.

We were surprised to find that the p53-dependent regulation of murine *Rbl2*/p130 relies on a cluster of p53 response elements within rapidly evolving Fuzzy Tandem Repeats (FTRs). This led us to identify two other murine p53 target genes regulated *via* FTRs containing p53 response elements: *Ncoa1* and *Klhl26* (Figure 2 and Figure 3). Both genes were found after BLAST searches over the mouse genome and expression assays in MEFs. It remains possible that other murine p53 targets regulated *via* FTRs escaped our search, because they are not expressed in MEFs and/or because BLAST was not programmed to detect FTRs. Although *mreps* is more capable of detecting FTRs, it detects repeats of any sequence, and thus cannot be used to search for repeats containing p53 REs over an entire genome. In fact, most programs for *ab initio* detection of repeats are unable to cope with a high level of sequence divergence between long (>24 nt) repeats [32]. In other words, fuzzy tandem repeats are difficult to detect over an entire genome due to their inherent fuzziness. Thus, the design of improved methods to find additional p53 targets regulated *via* fuzzy tandem repeats is an important goal. Interestingly, within tandem repeats, the number of clustered p53 REs with high Consite scores appears to correlate with the amount of p53 bound to the cluster, according to ChIP assays. Indeed, the cluster at the *Klhl26* locus, with 5 high scoring p53 REs, is strongly bound by p53 (Table S4, Figure 3D), whereas the cluster at the *Ncoa1* locus, containing only 1 high scoring p53 RE, appears weakly or transiently bound (Table S2, Figure 2D), and the cluster at *Rbl2* ranges in between for both criteria (Table S1, Figure 1F). The identification of a larger number of genes regulated *via* p53 RE-containing FTRs would allow to test this possible correlation further.

Importantly, we found that the fuzzy tandem repeats are poorly conserved in evolution, so that the p53-dependent regulation of the 3 genes appears only partially conserved among rodents, and does not operate in humans. Two out of the 3 identified genes are known to be relevant to cancer biology: *Rbl2* is frequently lost in a variety of human cancers and acts as a *bona fide* tumor suppressor in mouse models (e.g. [30]) and *Ncoa1* belongs to a family of transcription co-activators often deregulated in human tumors [33]. Regarding *Klhl26*, its function is currently unknown, but it was found mutated in head and neck squamous cell or ovarian carcinomas [34], [35]. Such a poor conservation in the regulation of genes apparently directly relevant to p53 tumor suppressive functions may seem surprising. However, it is possible that partial functional redundancy, or compensatory mechanisms, buffer the phenotypic consequences of diverging p53 target gene repertoires among mammals. The evolution of microRNA regulatory networks among mammalian species might provide such buffering, as recently suggested for the microRNA miR-125b [36].

Unstable tandem repeats in promoters confer transcriptional evolvability in yeasts [37], and evidence of similar mechanisms in other organisms is accumulating [38]. Our data indicate that the rapid evolution of fuzzy tandem repeats containing p53 REs may shape differences in p53 transcriptional networks among mammals. Recently, mice “humanized” to carry a human-specific SNP in the Mdm2 promoter allowed to demonstrate its importance on tumor onset [39]. The characterization of species-specific p53 target genes might help to define which genes should have their regulatory sequences humanized, with the aim of improving mouse models. Importantly, the possible role of fuzzy tandem repeats in shaping the target gene repertoire of other mammalian transcription factors deserves further investigation.

## Methods

### Cells and tissue cell culture reagents

Primary mouse embryonic fibroblasts were isolated from 13.5 day embryos from p53^+/−^ intercrosses, and cultured in a 5% CO_2_ and 3% O_2_ incubator for a maximum of 5 passages in DMEM Glutamax (Gibco), completed with 15% FBS (Biowest), 100 mM 2-mercaptoethanol (Millipore), 10 µM Non Essential Amino-Acids and Penicillin/Streptomycin (Gibco). Human fibroblasts from the lung (MRC5) or the foreskin (BJ) were purchased from the American Tissue Culture Cell Collection and cultured in a 5% CO_2_ and 3% O_2_ regulated incubator in MEM (Gibco), completed with 10% FBS, 2 mM L-Glutamine (Gibco), 1 mM Pyruvate, 10 µM Non Essential Amino-Acids and Penicillin/Streptomycin. HCA2 foreskin fibroblasts, originally prepared by O. Pereira, were grown like BJ cells. Primary rat embryonic fibroblasts, isolated from 14.5 days WT embryos, were a kind gift from O. Brison. All cells were treated with 0.5 µg/ml doxorubicin or with 10 µM Nutlin 3a for 24 h before RNA or protein extractions.

### Quantitative RT–PCR

Total RNA was extracted using NucleoSpin RNA II (Macherey-Nagel) and reverse transcribed with Superscript III First-Strand Synthesis Supermix (Invitrogen). Real-time quantitative PCR was performed on an ABi Prism 7500 system using Power SYBR Green master mix (ABi). Primers for detecting cDNA sequence of mouse, human or rat p21, p130, Ncoa1, Klhl26 and controls Rplp0 and PPIA were designed with Primer3 Input (version 0.4.0). All mRNA expression levels were normalized to both Rplp0 and PPIA. Primer sequences are listed in Table S5.

### Western blots

Protein detection by immunoblotting was performed using antibodies raised against p53 (CM5, Novocastra), p130 (C20, Santa-Cruz), GAPDH (mAb 374, Millipore), Ncoa1 (M-341, Santa Cruz), Klhl26 (C-14, Santa Cruz) and actin (A2066, Sigma). Chemiluminescence revelation of western blots was achieved with the SuperSignal substrates (Perbio, France).

### *In silico* search for putative p53 response elements

We first used a positional frequency matrix (PFM) for p53 response elements [19], modified to take varying spacer lengths (0–13 bp) into account (Figure S2), with the Consite software (http://asp.ii.uib.no:8090/cgi-bin/CONSITE/consite) to calculate the average score of p53 REs from known p53 target genes [17]: a mean value (M) of 11.7 was found, with a standard deviation (SD) of 1.2. We then used the same PFM to search for putative p53 REs at the *Rbl2*, *Ncoa1* and *Klhl26* loci, and considered motifs with a CNWGNNN(0–13)NNNCNWR core sequence and a Consite score >10.5 as putative REs. Putative REs were plotted against the map as lollipops and included in Tables S1, S2, S3, S4, S5, S6, S7, S8, with greytones according to their score: white for scores between 10.5 and 12.9 (M+/−SD), black for scores >15.3 (M+3 SD), and grey for scores between 12.9 and 15.3.

### *In silico* search for clustered p53 half-sites

Basic local alignment tool (BLAST) searches were performed using MouseBLAST (running WU-BLAST 2.0) from the Mouse Genome Informatics website (www.informatics.jax.org), with the BLASTN and Repeatmasker/rodents options. BAC clones identified as containing significant homology to clusters were then analyzed with Map Viewer from the National Center for Biotechnology Information (NCBI) website (www.ncbi.nlm.nih.gov/projects/mapview) to check if they contained genes. The DNA sequences of BACs with genes were retrieved using the NCBI clone registry and p53 half-site clusters were precisely mapped relative to genes by using Ensembl mouse gene annotations (www.ensembl.org/Mus_musculus/Info/Index).

### Sequence analysis with *mreps*

We used *mreps* (http://bioinfo.lifl.fr/mreps/mreps.php) with default settings, and searched for repeats with error rates <0.2. This revealed fuzzy tandem repeats for the 3 murine clusters. When homologous human sequences were analyzed with the same criteria, *mreps* failed to detect fuzzy repeats.

### Luciferase expression assays

To construct the p53 RE reporter plasmids, we cloned full promoters upstream of the firefly luciferase gene in a pGL3-basic vector (Promega), and partial promoter or intronic sequences upstream of a SV40-minimal promoter before the firefly luciferase gene (in a vector called below pGL3-PromMini or EmRep). Primers used to construct the reporter plasmids are listed in Table S6. For each experiment, 10^6^ exponentially growing p53^−/−^ cells were nucleofected (using the Lonza MEF2 nucleofector kit) by 3 µg of a p53 RE-firefly luciferase reporter plasmid; 3 µg of the same reporter plasmid and 3 µg of a WT p53 expression plasmid; or 3 µg of the same reporter plasmid and 3 µg of a p53^R270H^ expression plasmid. For all points, data were normalized by adding 30 ng of renilla luciferase expression plasmid (pGL4.73, Promega). Nucleofected cells were allowed to grow for 24 h, then trypsinized, resuspended in 75 µl culture medium and transferred into a well of an optical 96 well plate (Nunc). The dual-glo luciferase assay system (Promega) was used according to the manufacturer's protocol to lyse the cells and read firefly and renilla luciferase signals.

### Chromatin immunoprecipitation

ChIP analysis was performed essentially as described [40]. In brief, adherent p53^+/+^ and p53^−/−^ MEFs were treated with doxorubicin (0.5 µg/ml) for 24 h. Cellular proteins from 10^8^ cells were crosslinked to chromatin with 1% formaldehyde for 10 min at 25°C. p53-DNA complexes were immunoprecipitated from total extracts by using 50 µg of rabbit polyclonal p53 antibody (FL-393, Santacruz) and 500 µg of sonicated chromatin. Rabbit IgG (Abcam) was used for control precipitation. Quantitative PCR was performed on an Applied Biosystems 7500 instrument using Power SYBR Green master mix (ABi). Primer sequences are reported in Table S7.

### Whole-body gamma-irradiation

Age-matched mice were irradiated at 6–8 weeks with a Cs gamma-irradiator with 2.9 Gy/min at a dose of 10 Gy. Mice were sacrificed 3 h later and organs were recovered, then total RNA was extracted using Trizol (Invitrogen) and quantified as before. Experiments were performed according to IACUC regulations.

## Supporting Information

Figure S1 Whole-body irradiation leads to *Rbl2/*p130 induction in tissues of wild-type but not p53^−/−^ mice. Age-matched 6–8 weeks old wild-type (WT) and p53^−/−^ (KO) mice were left untreated or submitted to 10 Gy whole-body gamma-irradiation (irr.), before RNA extraction and real-time PCR quantification. Results are from 4 WT and 4 p53^−/−^ mice.
(TIF)

Figure S2 Matrices used for the search of candidate p53 Response Elements. (A) The positional frequency matrix used to search for candidate p53 Response Elements is shown. For each of the 20 positions, the percentage of occurrence for nucleotides A, C, G, and T is represented. The matrix comes from experimental data by Smeenk et al. [19]. (B) Matrices with varying spacer length used in our Consite analyses. The spacers of varying length were derived from the matrix in (A) by adding 0–13 equiprobable values after the first half-site. Five out of the 14 matrices used are shown as examples (numbers in parentheses indicate spacer length).
(TIF)

Figure S3 The cluster of p53REs in murine *Rbl2* mediates a p53-dependent regulation. Luciferase was measured in p53^−/−^ MEFs transfected with an empty reporter plasmid (EmRep, a plasmid with a SV40-minimal promoter before the firefly luciferase gene), or with the same plasmid and an expression vector for WT p53 (EmRep+p53WT) or mutant p53 (EmRep+p53R270H). Likewise, ClusRep, a reporter plasmid in which a 163 bp-long fragment encompassing the *Rbl2* cluster of p53REs was cloned upstream the SV40-minimal promoter, was transfected into p53^−/−^ MEFs alone (ClusRep), or with expression vectors for WT (ClusRep+p53WT) or mutant p53 (ClusRep+p53R270H), and luciferase activity was measured. Results, from 2 independent experiments, were normalized to control renilla luciferase, then a value of 1 was assigned to luciferase in cells transfected with reporter plasmids alone.
(TIF)

Figure S4 Detailed results of the sequence analysis of the murine *Rbl2* cluster with *mreps*. Positions are relative to the transcription start site. Period designates the length of a repeat, exponent the number of its occurrences, size the total length, and error rate the relative weight of mismatches. Alignments with error rates <0.2 are shown.
(TIF)

Figure S5 A p53-dependent increase in Ncoa1 and Klhl26 protein levels. p53^−/−^ and WT MEFs were left untreated or treated with doxorubicin (Dox), then protein extracts were immunoblotted with antibodies to Ncoa1, Klhl26, p53 and actin. A clear p53-dependent induction of Ncoa1 is visible. Endogenous Klhl26 levels were barely visible, but were detected more easily in WT stressed cells.
(TIF)

Figure S6 Detailed results of the sequence analysis of the murine *Ncoa1* cluster with *mreps*. Positions are relative to the transcription start site. Results are presented as in Figure S4.
(TIF)

Figure S7 Detailed results of the sequence analysis of the murine *Klhl26* cluster with *mreps*. Positions are relative to the transcription start site. Results are presented as in Figure S4.
(TIF)

Figure S8 Sequences of clustered putative p53 half-sites from 11 mammalian *Rbl2* loci. Clustered putative p53 binding half-sites (containing 0–3 mismatches with the consensus) were searched for in *Rbl2* sequences from 11 mammalian species and plotted as follows: putative half-sites are in bold (with matches in capital letters and mismatches in lowercase); perfect half-sites are boxed; half-sites with a single mismatch are underlined; half-sites with a deletion or insertion in the CWWG core are within parentheses. Numbers are relative to the transcription start sites (mouse, rat, dog, cattle, rhesus monkey, gibbon, chimp, human) or the translation start sites (rabbit, horse, elephant).
(TIF)

Figure S9 Sequences of clustered putative p53 half-sites from 7 mammalian *Klhl26* loci. Clustered putative p53 binding half-sites in *Klhl26* sequences from 7 mammalian species were searched for and plotted as in Figure S8. Numbers are relative to the transcription start sites.
(TIF)

Table S1 Consite analysis of Murine and Human *Rbl2* loci. Candidate p53 REs are listed, with spacer sequences in lowercase. Half-sites from candidate p53 REs mapping in the cluster region are in bold type, those from p53 REs outside of the cluster are in italics (see also Figure 1). Gtn: Greytone assigned according to score, as for lollipops in Figure 1.
(DOC)

Table S2 Consite analysis of clustered putative p53 half-sites upstream murine *Ncoa1*. Candidate p53 REs are listed as in Table S1.
(DOC)

Table S3 Sequences of the 128 p53 RE motifs used in whole genome BLAST searches. The 128 motifs are likely to be the most frequent p53 response elements, because they are composed of nucleotides present, at each position, in at least 34% of p53-bound REs (according to the PFM, see Figure S2). For each p53 RE, a nucleotide that differs from that at the same position in the first motif is highlighted in red.
(DOC)

Table S4 Consite analysis of clustered putative p53 half-sites in murine *Klhl26*. Candidate p53 REs are listed as in Table S1.
(DOC)

Table S5 Primers used in quantitative RT-PCR experiments.
(DOC)

Table S6 Primers and methods for the construction of luciferase reporter plasmids.
(DOC)

Table S7 Primers used in ChIP experiments.
(DOC)
