# Supplementary figures and images for: Fuzzy Tandem Repeats Containing p53 Response Elements May Define Species-Specific p53 Target Genes
Source: PLoS Genet. 2012 Jun 28;8(6):e1002731. doi: 10.1371/journal.pgen.1002731 (PMC3386156; doi:10.1371/journal.pgen.1002731)

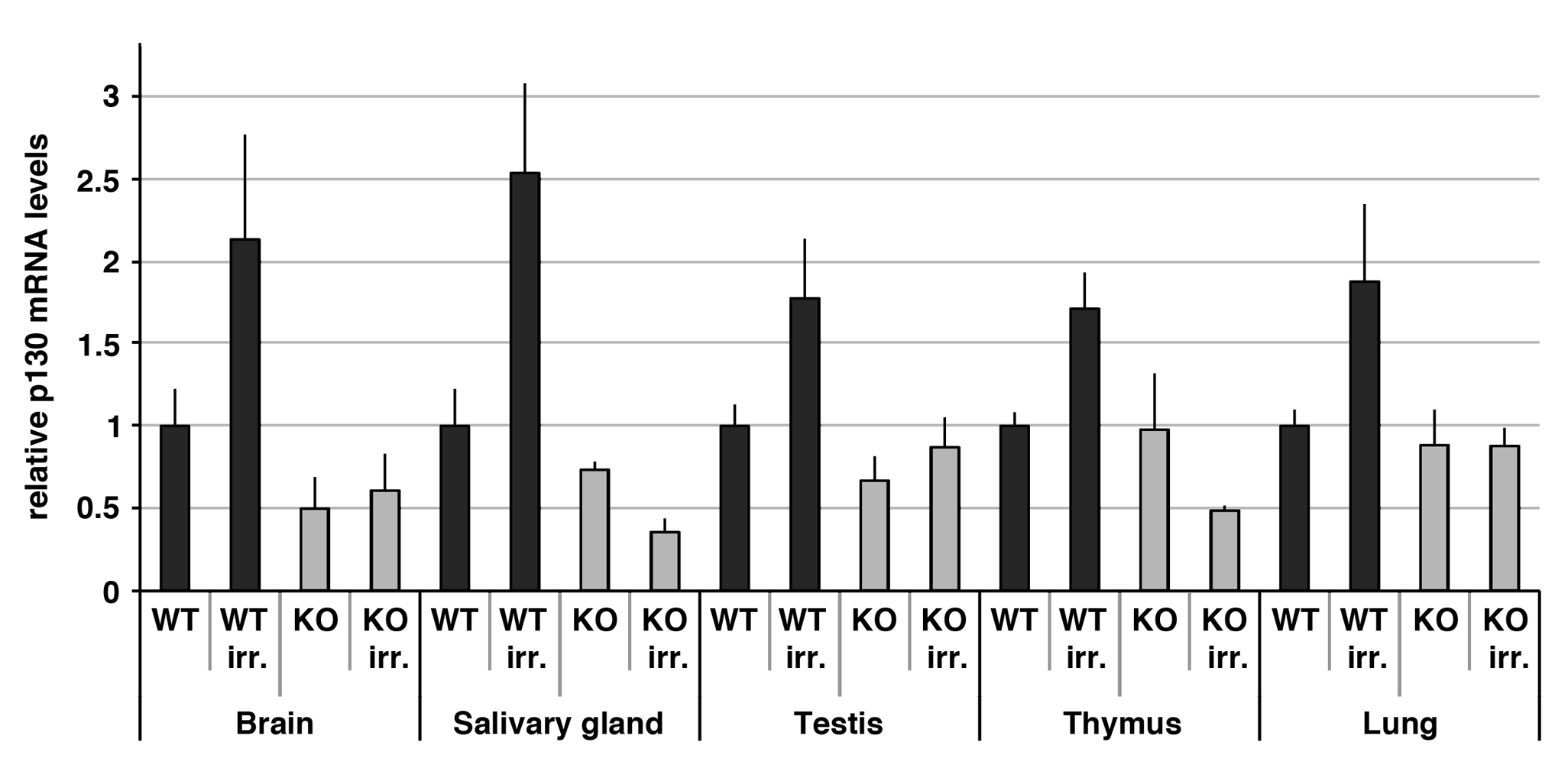

Supplement: Figure S1 — Whole-body irradiation leads to Rbl2/p130 induction in tissues of wild-type but not p53−/− mice. Age-matched 6–8 weeks old wild-type (WT) and p53−/− (KO) mice were left untreated or submitted to 10 Gy whole-body gamma-irradiation (irr.), before RNA extraction and real-time PCR quantification. Results are from 4 WT and 4 p53−/− mice. (TIF) [file pgen.1002731.s001.tif]

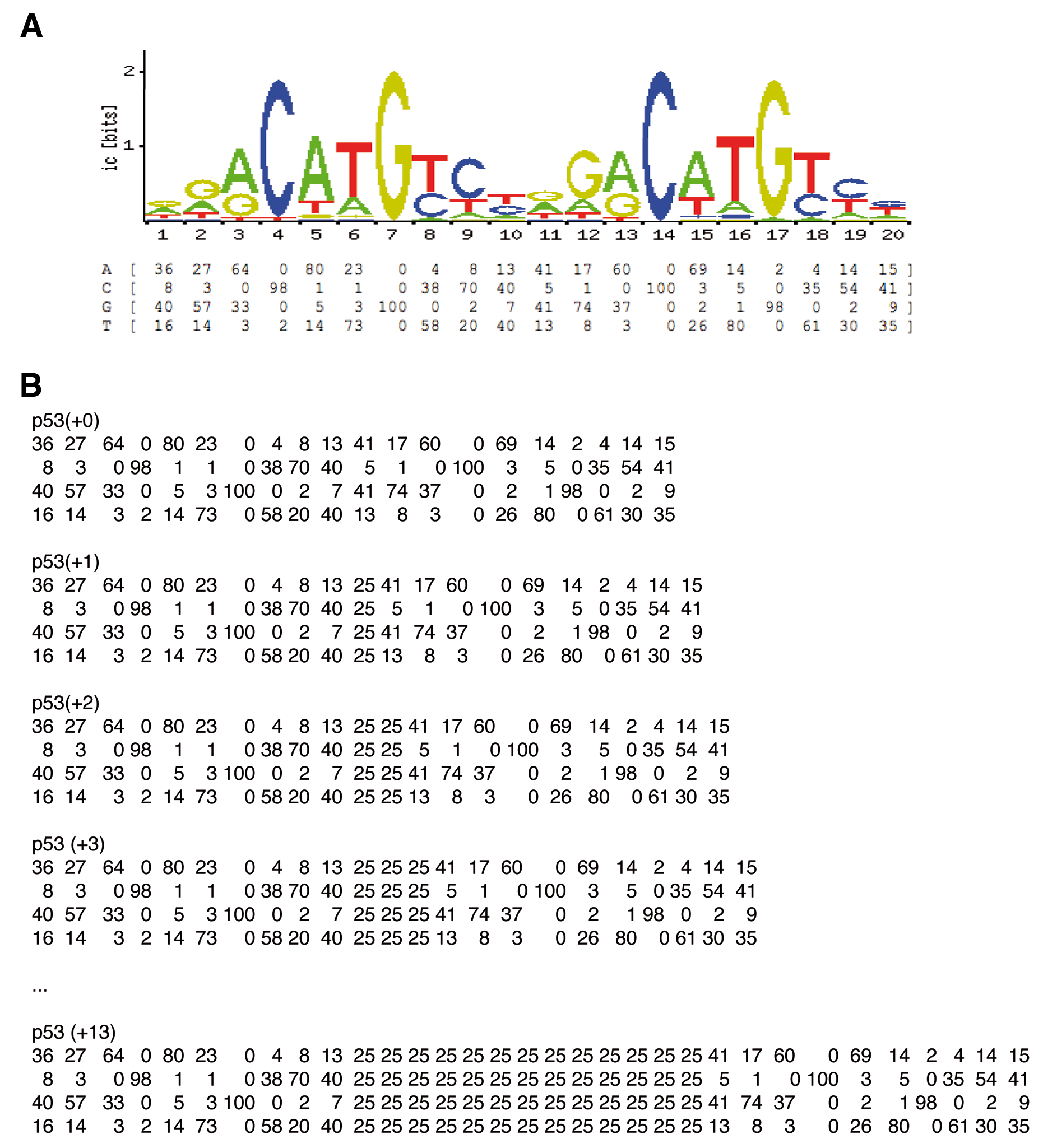

Supplement: Figure S2 — Matrices used for the search of candidate p53 Response Elements. (A) The positional frequency matrix used to search for candidate p53 Response Elements is shown. For each of the 20 positions, the percentage of occurrence for nucleotides A, C, G, and T is represented. The matrix comes from experimental data by Smeenk et al. [19]. (B) Matrices with varying spacer length used in our Consite analyses. The spacers of varying length were derived from the matrix in (A) by adding 0–13 equiprobable values after the first half-site. Five out of the 14 matrices used are shown as examples (numbers in parentheses indicate spacer length). (TIF) [file pgen.1002731.s002.tif]

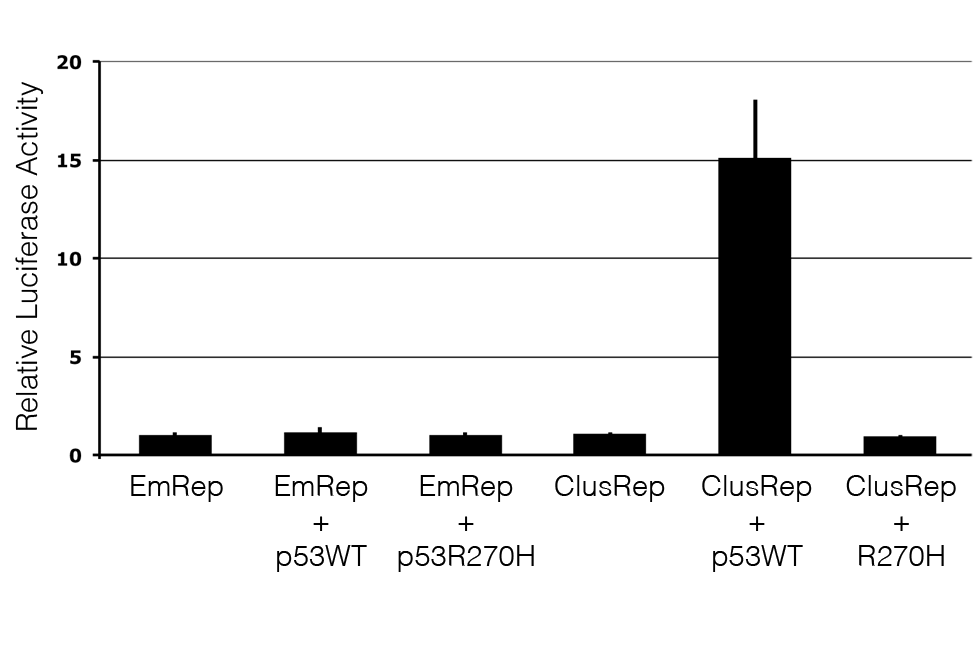

Supplement: Figure S3 — The cluster of p53REs in murine Rbl2 mediates a p53-dependent regulation. Luciferase was measured in p53−/− MEFs transfected with an empty reporter plasmid (EmRep, a plasmid with a SV40-minimal promoter before the firefly luciferase gene), or with the same plasmid and an expression vector for WT p53 (EmRep+p53WT) or mutant p53 (EmRep+p53R270H). Likewise, ClusRep, a reporter plasmid in which a 163 bp-long fragment encompassing the Rbl2 cluster of p53REs was cloned upstream the SV40-minimal promoter, was transfected into p53−/− MEFs alone (ClusRep), or with expression vectors for WT (ClusRep+p53WT) or mutant p53 (ClusRep+p53R270H), and luciferase activity was measured. Results, from 2 independent experiments, were normalized to control renilla luciferase, then a value of 1 was assigned to luciferase in cells transfected with reporter plasmids alone. (TIF) [file pgen.1002731.s003.tif]

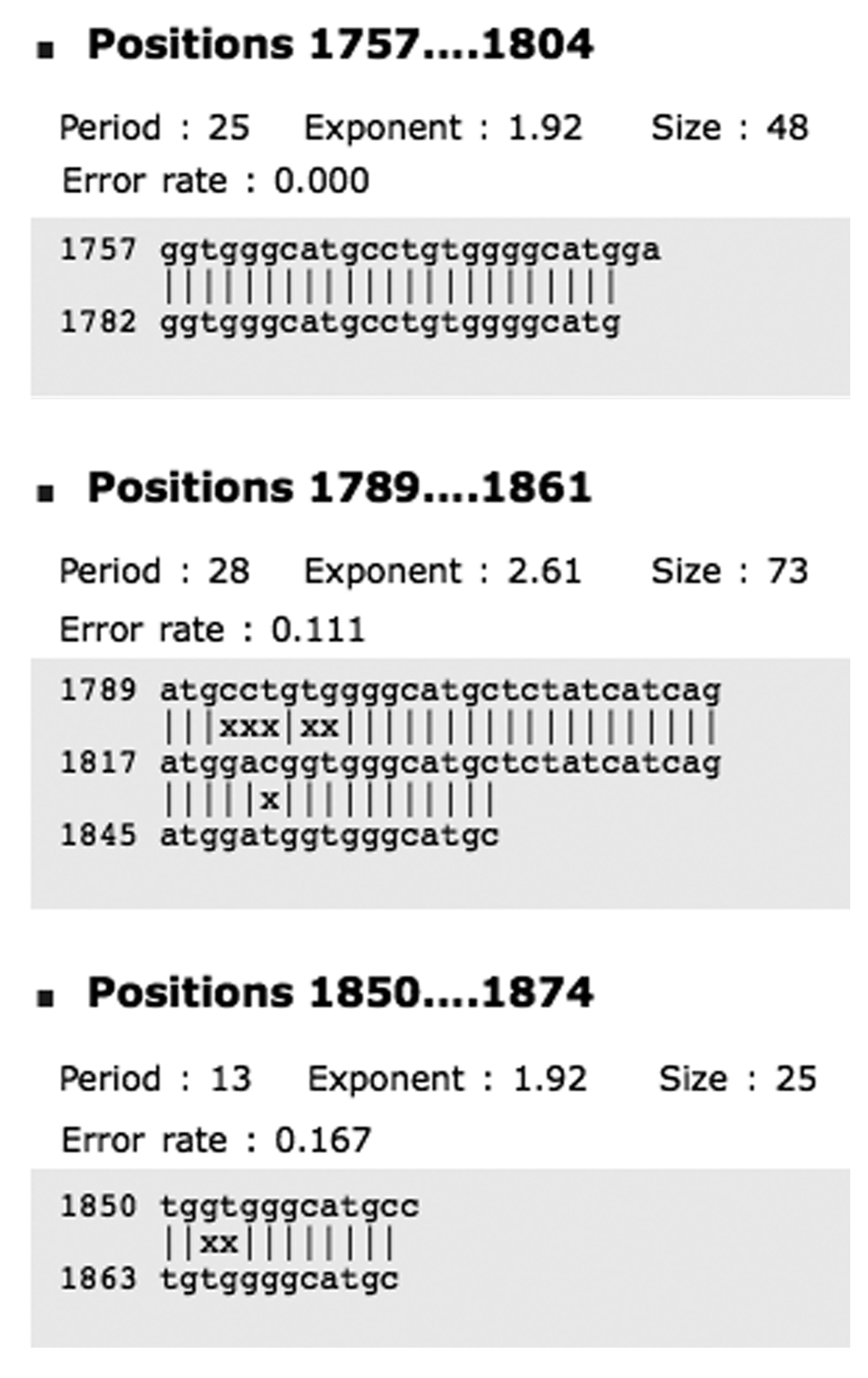

Supplement: Figure S4 — Detailed results of the sequence analysis of the murine Rbl2 cluster with mreps. Positions are relative to the transcription start site. Period designates the length of a repeat, exponent the number of its occurrences, size the total length, and error rate the relative weight of mismatches. Alignments with error rates <0.2 are shown. (TIF) [file pgen.1002731.s004.tif]

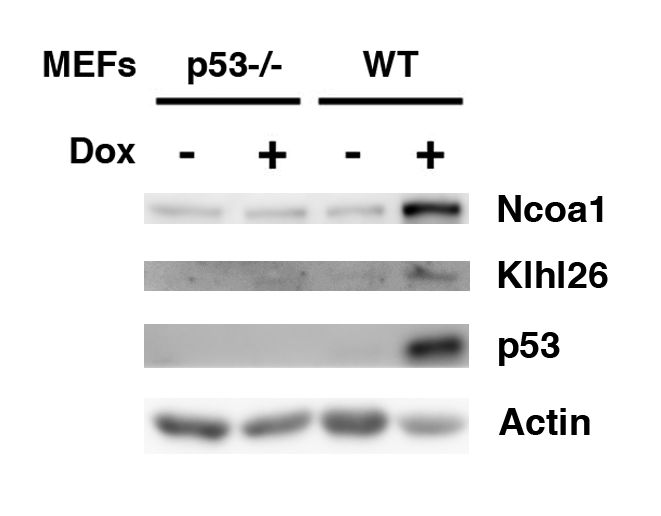

Supplement: Figure S5 — A p53-dependent increase in Ncoa1 and Klhl26 protein levels. p53−/− and WT MEFs were left untreated or treated with doxorubicin (Dox), then protein extracts were immunoblotted with antibodies to Ncoa1, Klhl26, p53 and actin. A clear p53-dependent induction of Ncoa1 is visible. Endogenous Klhl26 levels were barely visible, but were detected more easily in WT stressed cells. (TIF) [file pgen.1002731.s005.tif]

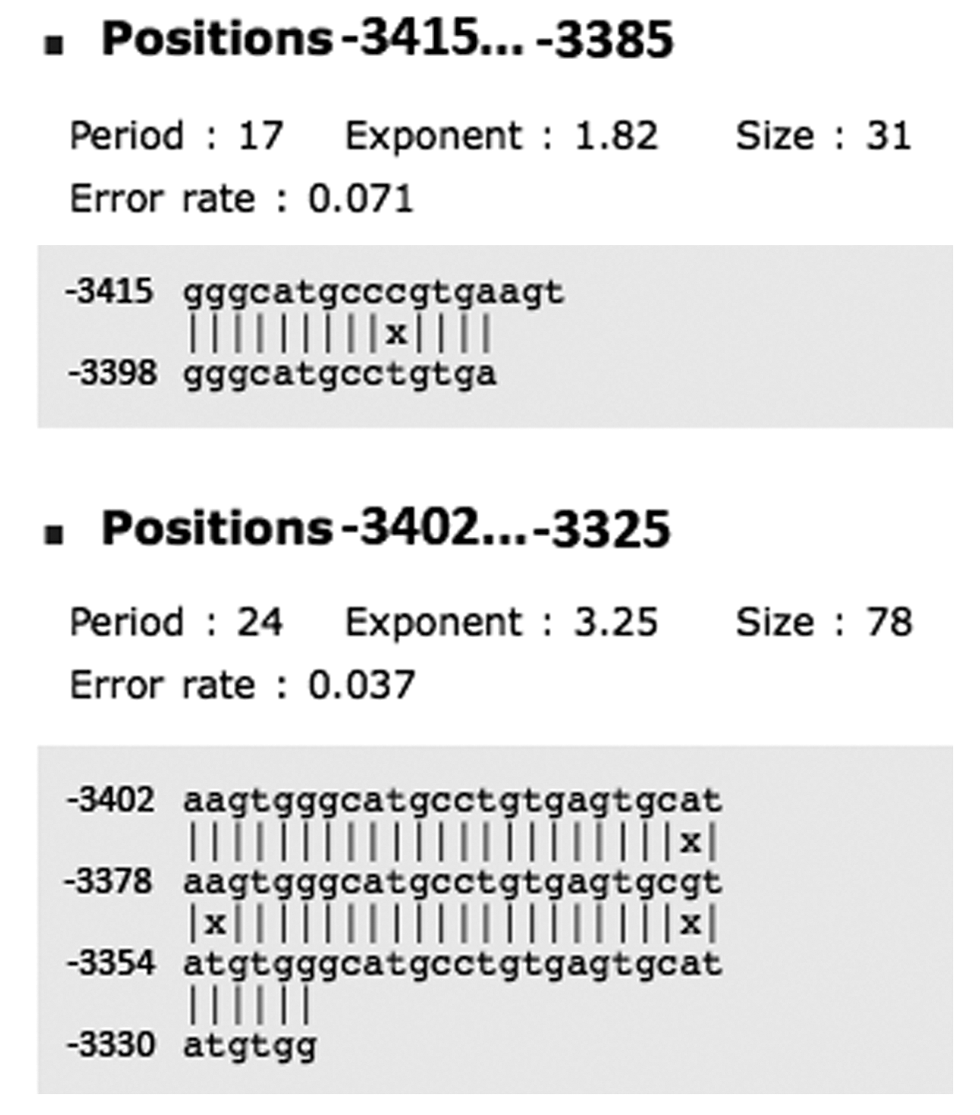

Supplement: Figure S6 — Detailed results of the sequence analysis of the murine Ncoa1 cluster with mreps. Positions are relative to the transcription start site. Results are presented as in Figure S4. (TIF) [file pgen.1002731.s006.tif]

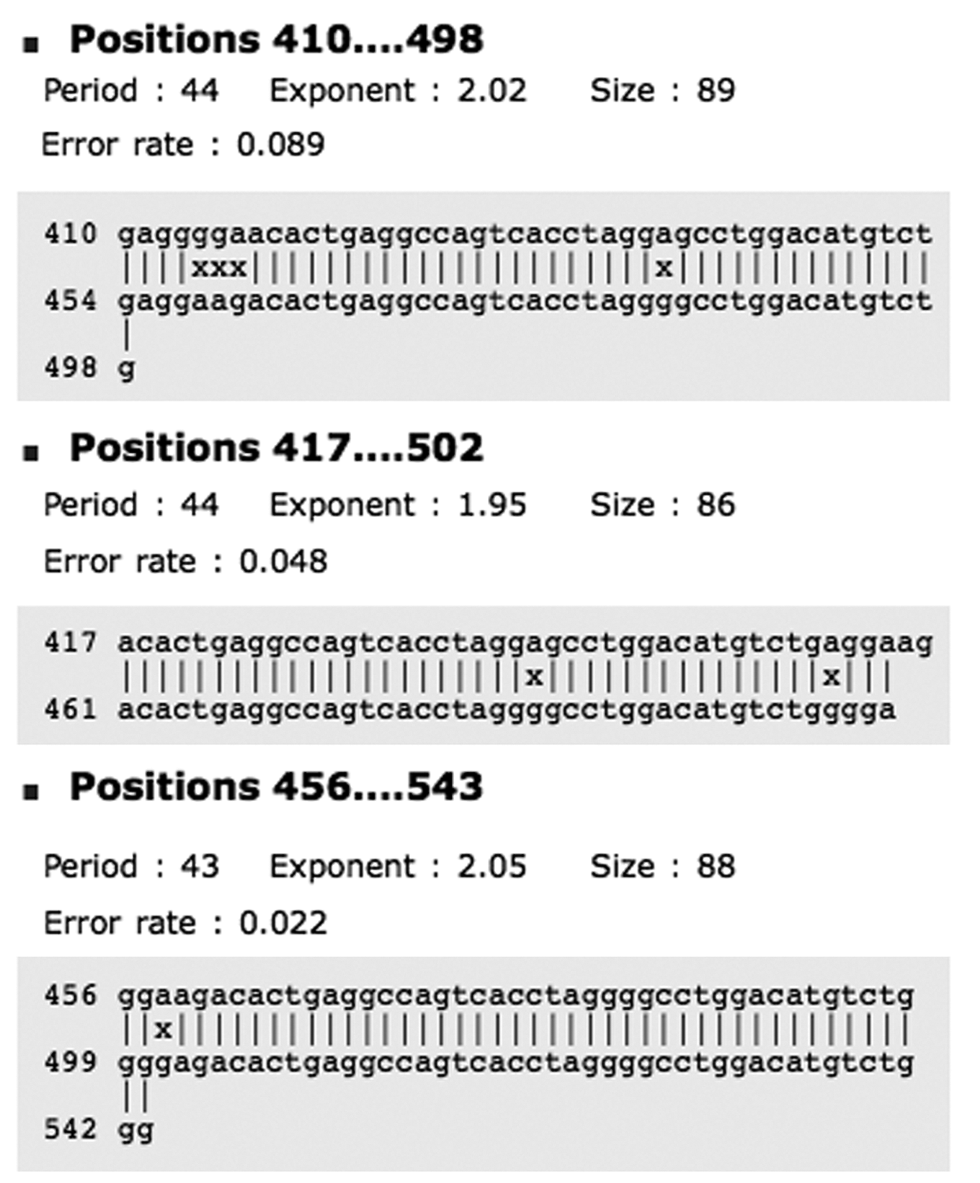

Supplement: Figure S7 — Detailed results of the sequence analysis of the murine Klhl26 cluster with mreps. Positions are relative to the transcription start site. Results are presented as in Figure S4. (TIF) [file pgen.1002731.s007.tif]

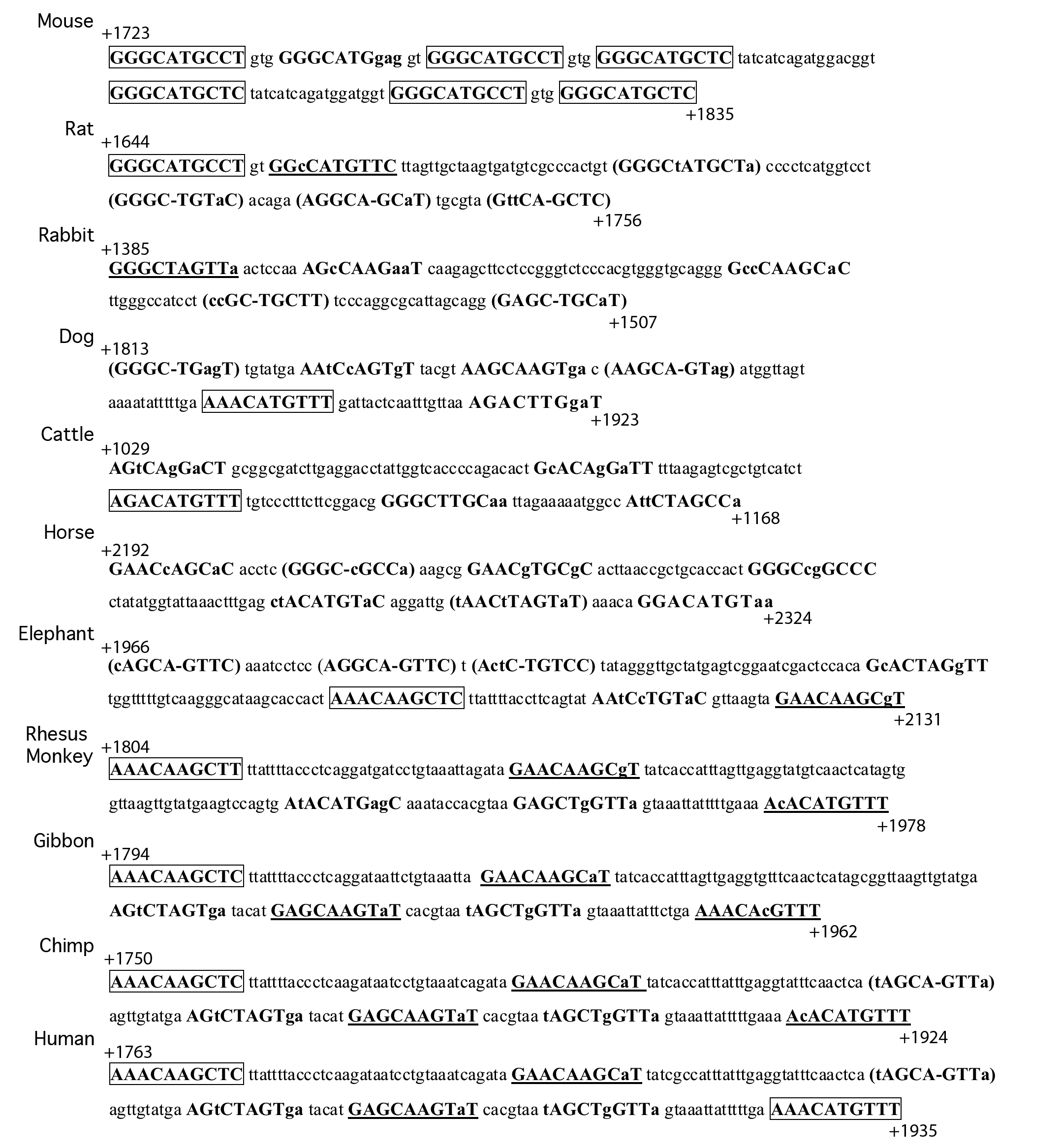

Supplement: Figure S8 — Sequences of clustered putative p53 half-sites from 11 mammalian Rbl2 loci. Clustered putative p53 binding half-sites (containing 0–3 mismatches with the consensus) were searched for in Rbl2 sequences from 11 mammalian species and plotted as follows: putative half-sites are in bold (with matches in capital letters and mismatches in lowercase); perfect half-sites are boxed; half-sites with a single mismatch are underlined; half-sites with a deletion or insertion in the CWWG core are within parentheses. Numbers are relative to the transcription start sites (mouse, rat, dog, cattle, rhesus monkey, gibbon, chimp, human) or the translation start sites (rabbit, horse, elephant). (TIF) [file pgen.1002731.s008.tif]

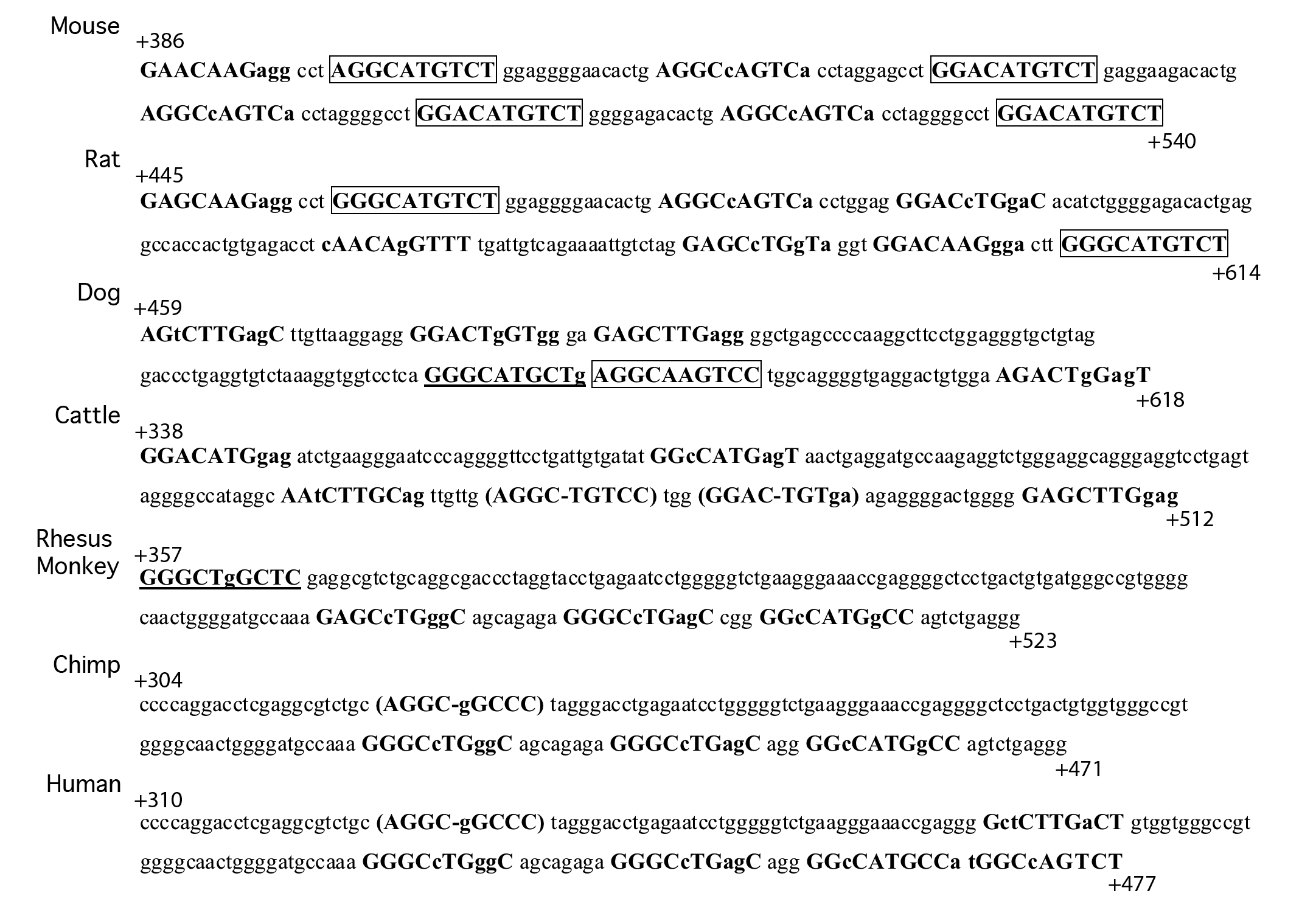

Supplement: Figure S9 — Sequences of clustered putative p53 half-sites from 7 mammalian Klhl26 loci. Clustered putative p53 binding half-sites in Klhl26 sequences from 7 mammalian species were searched for and plotted as in Figure S8. Numbers are relative to the transcription start sites. (TIF) [file pgen.1002731.s009.tif]
