# Supplementary material for: Fuzzy Tandem Repeats Containing p53 Response Elements May Define Species-Specific p53 Target Genes
Source: PLoS Genet. 2012 Jun 28;8(6):e1002731. doi: 10.1371/journal.pgen.1002731 (PMC3386156; doi:10.1371/journal.pgen.1002731)
Supplement: Table S1 — Consite analysis of Murine and Human Rbl2 loci. Candidate p53 REs are listed, with spacer sequences in lowercase. Half-sites from candidate p53 REs mapping in the cluster region are in bold type, those from p53 REs outside of the cluster are in italics (see also Figure 1). Gtn: Greytone assigned according to score, as for lollipops in Figure 1. (DOC) [file pgen.1002731.s010.doc]

**Table S1. Consite analysis of Murine and Human *Rbl2* loci.**

| **PFM (+spacer)** | **Position Start /TSS** | | **Position End /TSS** | **Sequence** | | | | **Consite score** | | **Gtn** | |
| --- | --- | --- | --- | --- | --- | --- | --- | --- | --- | --- | --- |
| **Murine *Rbl2*** | | | | |  |  |  | |  |  |  |
| p53 (+13) | *-2523* | | *-2491* | *TGGCATGTAA*tcaagaatatatc*AAACATGCCA* | | | | *15.543* | |  | |
| p53 (+5) | *+957* | | *+981* | *AAACATGGCA*gcttt*AGACCTGTGT* | | | | *11.436* | |  | |
| p53 (+3) | **+1760** | | **+1782** | **GGGCATGCCT**gtg**GGGCATGGAG** | | | | **15.429** | |  | |
| p53 (+2) | **+1773** | | **+1794** | **GGGCATGGAG**gt**GGGCATGCCT** | | | | **13.850** | |  | |
| p53 (+3) | **+1785** | | **+1807** | **GGGCATGCCT**gtg**GGGCATGCTC** | | | | **22.233** | |  | |
| p53 (+3) | **+1854** | | **+1876** | **GGGCATGCCT**gtg**GGGCATGCTC** | | | | **22.233** | |  | |
| **Human *Rbl2*** | |  |  |  | | | |  | |  | |
| p53(+5) | *-5923* | | *-5899* | *AGACATGAAA*cagtc*AAACATGTAA* | | | | *13.272* | |  | |
| p53 (+3) | *+708* | | *+730* | *GGACAAGGGG*agg*GGACTTGCCC* | | | | *11.516* | |  | |

Candidate p53 REs are listed, with spacer sequences in lowercase. Half sites from candidate p53 REs mapping in the cluster region are in bold type, those from p53 REs outside of the cluster are in italics (see also Figure 1). Gtn : Greytone assigned according to score, as for lollipops in Figure 1.
