# Supplementary material for: Fuzzy Tandem Repeats Containing p53 Response Elements May Define Species-Specific p53 Target Genes
Source: PLoS Genet. 2012 Jun 28;8(6):e1002731. doi: 10.1371/journal.pgen.1002731 (PMC3386156; doi:10.1371/journal.pgen.1002731)
Supplement: Table S2 — Consite analysis of clustered putative p53 half-sites upstream murine Ncoa1. Candidate p53 REs are listed as in Table S1. (DOC) [file pgen.1002731.s011.doc]

**Table S2. Consite analysis of clustered putative p53 half sites upstream murine *Ncoa1*.**

| **PFM (+spacer)** | **Start /TSS** | **End /TSS** | | **Sequence** | | | **Consite score** | **Gtn** | | |
| --- | --- | --- | --- | --- | --- | --- | --- | --- | --- | --- |
| **Mouse *Ncoa1* cluster** | | |  | |  |  | | |  |  |
| p53(+7) | **-3415** | **-3389** | | **GGGCATGCCC**gtgaagt**GGGCATGCCT** | | | **22.888** |  | | |
| p53(+11) | **-3415** | **-3385** | | **GGGCATGCCC**gtgaagtgggc**ATGCCTGTGA** | | | **12.735** |  | | |
| p53(+10) | **-3394** | **-3363** | | **ATGCCTGTGA**gtgcataagt**GGGCATGCCT** | | | **11.379** |  | | |
| p53(+10) | **-3370** | **-3341** | | **ATGCCTGTGA**gtgcgtatgt**GGGCATGCCT** | | | **11.379** |  | | |
| p53(+4) | **-3348** | **-3327** | | **GGGCATGCCT**gtga**GTGCATATGT** | | | **13.090** |  | | |

Candidate p53 REs are listed as in Table S1.
