# Supplementary material for: Fuzzy Tandem Repeats Containing p53 Response Elements May Define Species-Specific p53 Target Genes
Source: PLoS Genet. 2012 Jun 28;8(6):e1002731. doi: 10.1371/journal.pgen.1002731 (PMC3386156; doi:10.1371/journal.pgen.1002731)
Supplement: Table S3 — Sequences of the 128 p53 RE motifs used in whole genome BLAST searches. The 128 motifs are likely to be the most frequent p53 response elements, because they are composed of nucleotides present, at each position, in at least 34% of p53-bound REs (according to the PFM, see Figure S2). For each p53 RE, a nucleotide that differs from that at the same position in the first motif is highlighted in red. (DOC) [file pgen.1002731.s012.doc]

**Table S3. Sequences of the 128 p53 RE motifs used in whole genome BLAST searches.**

GGACATGTCCAGACATGTCC

GGACATGTCCAGACATGTCT

GGACATGTCCAGACATGCCC

GGACATGTCCAGACATGCCT

GGACATGTCCAGGCATGTCC

GGACATGTCCAGGCATGTCT

GGACATGTCCAGGCATGCCC

GGACATGTCCAGGCATGCCT

GGACATGTCCGGACATGTCC

GGACATGTCCGGACATGTCT

GGACATGTCCGGACATGCCC

GGACATGTCCGGACATGCCT

GGACATGTCCGGGCATGTCC

GGACATGTCCGGGCATGTCT

GGACATGTCCGGGCATGCCC

GGACATGTCCGGGCATGCCT

GGACATGTCTAGACATGTCC

GGACATGTCTAGACATGTCT

GGACATGTCTAGACATGCCC

GGACATGTCTAGACATGCCT

GGACATGTCTAGGCATGTCC

GGACATGTCTAGGCATGTCT

GGACATGTCTAGGCATGCCC

GGACATGTCTAGGCATGCCT

GGACATGTCTGGACATGTCC

GGACATGTCTGGACATGTCT

GGACATGTCTGGACATGCCC

GGACATGTCTGGACATGCCT

GGACATGTCTGGGCATGTCC

GGACATGTCTGGGCATGTCT

GGACATGTCTGGGCATGCCC

GGACATGTCTGGGCATGCCT

GGACATGCCCAGACATGTCC

GGACATGCCCAGACATGTCT

GGACATGCCCAGACATGCCC

GGACATGCCCAGACATGCCT

GGACATGCCCAGGCATGTCC

GGACATGCCCAGGCATGTCT

GGACATGCCCAGGCATGCCC

GGACATGCCCAGGCATGCCT

GGACATGCCCGGACATGTCC

GGACATGCCCGGACATGTCT

GGACATGCCCGGACATGCCC

GGACATGCCCGGACATGCCT

GGACATGCCCGGGCATGTCC

GGACATGCCCGGGCATGTCT

GGACATGCCCGGGCATGCCC

GGACATGCCCGGGCATGCCT

GGACATGCCTAGACATGTCC

GGACATGCCTAGACATGTCT

GGACATGCCTAGACATGCCC

GGACATGCCTAGACATGCCT

GGACATGCCTAGGCATGTCC

GGACATGCCTAGGCATGTCT

GGACATGCCTAGGCATGCCC

GGACATGCCTAGGCATGCCT

GGACATGCCTGGACATGTCC

GGACATGCCTGGACATGTCT

GGACATGCCTGGACATGCCC

GGACATGCCTGGACATGCCT

GGACATGCCTGGGCATGTCC

GGACATGCCTGGGCATGTCT

GGACATGCCTGGGCATGCCC

GGACATGCCTGGGCATGCCT

AGACATGTCCAGACATGTCC

AGACATGTCCAGACATGTCT

AGACATGTCCAGACATGCCC

AGACATGTCCAGACATGCCT

AGACATGTCCAGGCATGTCC

AGACATGTCCAGGCATGTCT

AGACATGTCCAGGCATGCCC

AGACATGTCCAGGCATGCCT

AGACATGTCCGGACATGTCC

AGACATGTCCGGACATGTCT

AGACATGTCCGGACATGCCC

AGACATGTCCGGACATGCCT

AGACATGTCCGGGCATGTCC

AGACATGTCCGGGCATGTCT

AGACATGTCCGGGCATGCCC

AGACATGTCCGGGCATGCCT

AGACATGTCTAGACATGTCC

AGACATGTCTAGACATGTCT

AGACATGTCTAGACATGCCC

AGACATGTCTAGACATGCCT

AGACATGTCTAGGCATGTCC

AGACATGTCTAGGCATGTCT

AGACATGTCTAGGCATGCCC

AGACATGTCTAGGCATGCCT

AGACATGTCTGGACATGTCC

AGACATGTCTGGACATGTCT

AGACATGTCTGGACATGCCC

AGACATGTCTGGACATGCCT

AGACATGTCTGGGCATGTCC

AGACATGTCTGGGCATGTCT

AGACATGTCTGGGCATGCCC

AGACATGTCTGGGCATGCCT

AGACATGCCCAGACATGTCC

AGACATGCCCAGACATGTCT

AGACATGCCCAGACATGCCC

AGACATGCCCAGACATGCCT

AGACATGCCCAGGCATGTCC

AGACATGCCCAGGCATGTCT

AGACATGCCCAGGCATGCCC

AGACATGCCCAGGCATGCCT

AGACATGCCCGGACATGTCC

AGACATGCCCGGACATGTCT

AGACATGCCCGGACATGCCC

AGACATGCCCGGACATGCCT

AGACATGCCCGGGCATGTCC

AGACATGCCCGGGCATGTCT

AGACATGCCCGGGCATGCCC

AGACATGCCCGGGCATGCCT

AGACATGCCTAGACATGTCC

AGACATGCCTAGACATGTCT

AGACATGCCTAGACATGCCC

AGACATGCCTAGACATGCCT

AGACATGCCTAGGCATGTCC

AGACATGCCTAGGCATGTCT

AGACATGCCTAGGCATGCCC

AGACATGCCTAGGCATGCCT

AGACATGCCTGGACATGTCC

AGACATGCCTGGACATGTCT

AGACATGCCTGGACATGCCC

AGACATGCCTGGACATGCCT

AGACATGCCTGGGCATGTCC

AGACATGCCTGGGCATGTCT

AGACATGCCTGGGCATGCCC

AGACATGCCTGGGCATGCCT

The 128 motifs are likely to be the most frequent p53 response elements, because they are composed of nucleotides present, at each position, in at least 34% of p53-bound REs (according to the PFM, see Figure S2). For each p53 RE, a nucleotide that differs from that at the same position in the first motif is highlighted in red.
