# Supplementary material for: Fuzzy Tandem Repeats Containing p53 Response Elements May Define Species-Specific p53 Target Genes
Source: PLoS Genet. 2012 Jun 28;8(6):e1002731. doi: 10.1371/journal.pgen.1002731 (PMC3386156; doi:10.1371/journal.pgen.1002731)
Supplement: Table S4 — Consite analysis of clustered putative p53 half-sites in murine Klhl26. Candidate p53 REs are listed as in Table S1. (DOC) [file pgen.1002731.s013.doc]

**Table S4. Consite analysis of clustered putative p53 half sites in murine *Klhl26****.*

| **PFM (+spacer)** | **Start /TSS** | **End /TSS** | | **Sequence** | | | **Consite score** | | | **Gtn** | |
| --- | --- | --- | --- | --- | --- | --- | --- | --- | --- | --- | --- |
| **Mouse *Klhl26* cluster** | | |  | |  |  | |  |  | |  |
| p53(+3) | **+386** | **+408** | | **GAACAAGAGG**cct**AGGCATGTCT** | | | **13.093** | | |  | |
| p53(+11) | **+423** | **+453** | | **AGGCCAGTCA**cctaggagcct**GGACATGTCT** | | | **17.446** | | |  | |
| p53(+13) | **+444** | **+476** | | **GGACATGTCT**gaggaagacactg**AGGCCAGTCA** | | | **18.057** | | |  | |
| p53(+11) | **+467** | **+497** | | **AGGCCAGTCA**cctaggggcct**GGACATGTCT** | | | **17.446** | | |  | |
| p53(+12) | **+488** | **+519** | | **GGACATGTCT**ggggagacactg**AGGCCAGTCA** | | | **18.057** | | |  | |
| p53(+11) | **+510** | **+540** | | **AGGCCAGTCA**cctaggggcct**GGACATGTCT** | | | **17.446** | | |  | |

Candidate p53 REs are listed as in Table S1.
