# Supplementary material for: Fuzzy Tandem Repeats Containing p53 Response Elements May Define Species-Specific p53 Target Genes
Source: PLoS Genet. 2012 Jun 28;8(6):e1002731. doi: 10.1371/journal.pgen.1002731 (PMC3386156; doi:10.1371/journal.pgen.1002731)
Supplement: Table S5 — Primers used in quantitative RT-PCR experiments. (DOC) [file pgen.1002731.s014.doc]

| **Species** | **Name** | **Sequence** |
| --- | --- | --- |
| **mouse** | p21-F | GCAGACCAGCCTGACAGATTC |
|  | p21-R | TTCAGGGTTTTCTCTTGCAGAAG |
|  | p130-F | GCAGCTACCGCAGCATGAG |
|  | p130-R | AAGGCACATGCTAACCAATGAA |
|  | Ncoa1-F | GCAGCCAAGGAGTGATAGAGAAG |
|  | Ncoa1-R | ACAGTTCACAACAAAGAAAAATCCAT |
|  | Klhl26-F | tcgatgtggtcctcactgtc |
|  | Klhl26-R | cctcagcactgtaggcaaaa |
|  | Rplp0-F | CGACCTGGAAGTCCAACTAC |
|  | Rplp0-R | ATCTGCTGCATCTGCTTG |
|  | Ppia-F | TCTCCTTCGAGCTGTTTGCA |
|  | Ppia-R | CAGTGCTCAGAGCTCGAAAGTTT |
| **rat** | p21-F | AGGCAGACCAGCCTAACAGA |
|  | p21-R | TTCAGGGCTTTCTCTTGCAG |
|  | p130-F | CTACACGCTGGAGGGAAATG |
|  | p130-R | CTTCAGCAGTCCCTTTGCTC |
|  | Ncoa1-F | AACCCCGGCAGACTCTAAAT |
|  | Ncoa1-R | TTGCTGCAAACTGGTTCAAG |
|  | Klhl26-F | TCGATGTGGTCCTCACTGTC |
|  | Klhl26-R | CCAGTGAACATGGCCCTAAA |
|  | Rplp0-F | GAGGTGCTGGACATCACAGA |
|  | Rplp0-R | TGATGGAGTGAGGCACTGAG |
|  | Ppia-F | AGCATACAGGTCCTGGCATC |
|  | Ppia-R | TTCACCTTCCCAAAGACCAC |
| **human** | p21-F | tggagactctcagggtcgaaaa |
|  | p21-R | cggcgtttggagtggtagaa |
|  | p130-F | ccgcagcatgagcgaaa |
|  | p130-R | ggcacatgctaaccaatgaaga |
|  | Ncoa1-F | ATACACCAAAATCGGCAAGC |
|  | Ncoa1-R | TGTGCCAACATTTGAGCATT |
|  | Klhl26-F | GCCTGCAGCGACTACTTCA |
|  | Klhl26-R | TAGGCGAAGTCGATGATGTG |
|  | Rplp0-F | cttgtctgtggagacggattacac |
|  | Rplp0-R | tacgccaagaaggccttga |
|  | Ppia-F | catctgcactgccaagactga |
|  | Ppia-R | ttcatgccttctttcactttgc |

**Table S5. Primers used in quantitative RT-PCR experiments.**
