# Supplementary material for: Fuzzy Tandem Repeats Containing p53 Response Elements May Define Species-Specific p53 Target Genes
Source: PLoS Genet. 2012 Jun 28;8(6):e1002731. doi: 10.1371/journal.pgen.1002731 (PMC3386156; doi:10.1371/journal.pgen.1002731)
Supplement: Table S6 — Primers and methods for the construction of luciferase reporter plasmids. (DOC) [file pgen.1002731.s015.doc]

**Table S6. Primers and methods for the construction of luciferase reporter plasmids.**

| **Plasmid name** | **Primers** | **Sequence** | **Comments** |
| --- | --- | --- | --- |
| **I** | p130F-KpnI | gatacaggtaccttactaaagtggtttcac | Product cloned |
|  | p130R-BglII | gatacaagatctagcagcctgtcctgctac | in PGL3-basic |
| **II** | I1p130F-KpnI | gatacaggtaccttgtttttcagctgcctgtg | Product cloned |
|  | I1p130R-MluI | gatacaacgcgtcgtgtttggtttggaggact | in PGL3-PromMini |
| **III** |  |  | Sph I digestion of |
|  |  |  | plasmid II, religation |
| **IV** | NcoaF-SacI | gatacagagctcatacaatgtgtggaaaattattcatta | Product cloned |
|  | NcoaR-MluI | gatacaacgcgtaagtgctgtgcggtcatctt | in PGL3-PromMini |
| **V** | NcoaF’-SacI | gatacagagctcctcgaaaagctaatgttcttaccta | Product cloned |
|  | NcoaR-MluI | gatacaacgcgtaagtgctgtgcggtcatctt | in PGL3-PromMini |
| **VI** | Klhl26-F-MluI | gatacaacgcgtgagtcctacgcctctgttgg | Product cloned |
|  | Klhl26-R-XhoI | gatacactcgaggatcattcgggttgaggaaa | in PGL3-PromMini |
| **VII** | fuseKlhl26F | acaggtcctgatggcaggGAGGGGACACTGAGGCAA | Derived from plasmid |
|  | fuseKlhl26R | TTGCCTCAGTGTCCCCTCcctgccatcaggacctgt | VI by Fusion PCR |
| **ClusRep** | I1-p130F1-KpnI | gatacaggtaccgcctcatctgaggagtcacc | Product cloned |
|  | I1-p130R1-MluI | gatacaacgcgtgggcgacatcaattagcaac | in PGL3-PromMini |
