# Supplementary material for: Fuzzy Tandem Repeats Containing p53 Response Elements May Define Species-Specific p53 Target Genes
Source: PLoS Genet. 2012 Jun 28;8(6):e1002731. doi: 10.1371/journal.pgen.1002731 (PMC3386156; doi:10.1371/journal.pgen.1002731)
Supplement: Table S7 — Primers used in ChIP experiments. (DOC) [file pgen.1002731.s016.doc]

**Table S7. Primers used in ChIP experiments.**

| **Locus** | **Name** | **Sequence** |
| --- | --- | --- |
| **p130** | p130 ChIP F | TCTTGTTTGGCTGAGGCGTTC |
|  | p130 ChIP R | GGCAAGGGTCAACTGGGCTA |
| **Ncoa1** | Ncoa1 ChIP F | GGGGAGGGTGGAAAAGTACCA |
|  | Ncoa1 ChIP R | AAAGAAAAGACAACTGGTAAGCCAGA |
| **Klhl26** | Klhl26 ChIP F | CCCCAGCAGATCCATCATG |
|  | Klhl26 ChIP R | CAAATACATCCCGACCTCTCACA |
| **Irrelevant site** | Irr ChIP F | CCAAGGGAAGCCACATGGT |
|  | Irr ChIP R | ACCAGCCTTTCCATCTGCAT |
